# A Randomized, Double-Blind, Placebo-Controlled Trial of Adjunctive Metformin Therapy in Overweight/Obese Youth with Type 1 Diabetes

**DOI:** 10.1371/journal.pone.0137525

**Published:** 2015-09-14

**Authors:** Benjamin Udoka Nwosu, Louise Maranda, Karen Cullen, Lisa Greenman, Jody Fleshman, Nancy McShea, Bruce A. Barton, Mary M. Lee

**Affiliations:** 1 Division of Pediatric Endocrinology, Department of Pediatrics, University of Massachusetts Medical School, 55 Lake Avenue N, Worcester, Massachusetts, 01655, United States of America; 2 Department of Quantitative Health Sciences, University of Massachusetts Medical School, 55 Lake Avenue N, Worcester, Massachusetts, 01655, United States of America; University, ITALY

## Abstract

**Context:**

Insulin resistance has been proposed as one of the causes of poor glycemic control in overweight/obese youth with type 1 diabetes (T1D). However, the role of adjunctive metformin, an insulin sensitizer, on glycemic control in these patients is unclear.

**Objective:**

To compare the effect of metformin vs. placebo on hemoglobin A1c (HbA1c), total daily dose (TDD) of insulin, and other parameters in overweight/obese youth with T1D.

**Hypothesis:**

Adjunctive metformin therapy will improve glycemic control in overweight/obese youth with T1D.

**Design, Setting, and Participants:**

A 9-mo randomized, double-blind, placebo controlled trial of metformin and placebo in 28 subjects (13m/15f) of ages 10-20years (y), with HbA1c >8% (64 mmol/mol), BMI >85%, and T1D > 12 months was conducted at a university outpatient facility. The metformin group consisted of 15 subjects (8 m/ 7f), of age 15.0 ± 2.5 y; while the control group was made up of 13 subjects (5m/ 8f), of age 14.5 ± 3.1y. All participants employed a self-directed treat-to-target insulin regimen based on a titration algorithm of (-2)-0-(+2) units to adjust their long-acting insulin dose every 3rd day from -3 mo through +9 mo to maintain fasting plasma glucose (FPG) between 90–120 mg/dL (5.0–6.7 mmol/L). Pubertal maturation was determined by Tanner stage.

**Results:**

Over the course of the 9 months of observation, the between-treatment differences in HbA1c of 0.4% (9.85% [8.82 to 10.88] for placebo versus 9.46% [8.47 to 10.46] for metformin) was not significant (p = 0.903). There were non-significant reduction in fasting plasma glucose (189.4 mg/dL [133.2 to 245.6] for placebo versus 170.5 mg/dL [114.3 to 226.7] for metformin), (p = 0.927); total daily dose (TDD) of short-acting insulin per kg body weight/day(p = 0.936); and the TDD of long-acting insulin per kg body weight per day (1.15 units/kg/day [0.89 to 1.41] for placebo versus 0.90 units/kg/day [0.64 to 1.16] for metformin) (p = 0.221). There was no difference in the occurrence of hypoglycemia between the groups.

**Conclusions:**

This 9-month RCT of adjunctive metformin therapy in overweight and obese youth with T1D resulted in a 0.4% lower HbA1c value in the metformin group compared to the placebo group.

**Trial Registration:**

ClinicalTrial.gov NCT01334125

## Introduction

Obese/overweight youth with type 1 diabetes (T1D) often have suboptimal glycemic control [1]. Though insulin resistance has been proposed as one of the causes of this poor glycemic control, the role of adjunctive metformin, an insulin sensitizer, on glycemic control in these patients is unclear.

Metformin is a biguanide, which acts principally by increasing insulin sensitivity in the liver by inhibiting hepatic gluconeogenesis and thereby reducing hepatic glucose output [2]. Randomized controlled trials with metformin in adolescents with type 2 diabetes (T2D) reported a decrease in fasting plasma glucose concentration [3]. However, there have been conflicting reports from studies in adolescents with T1D [2, 4–7]. The benefit was transient in one study [7] and no decrease was found in another [4]. The main drawback of these studies was the small sample size and lack of reporting on long term benefit and safety of adjunctive therapy in many of them [8].

To address this question, we conducted a 12-month clinical trial consisting of a 3-month run-in-phase and a 9-month randomized, double-blind, placebo-controlled interventional period to determine the role of adjunctive metformin therapy on glycemic control in overweight/obese youth with T1D. We hypothesized that adjunctive metformin therapy would improve glycemic control in overweight/obese youth with T1D. The study’s objective was to compare the effect of adjunctive metformin vs. placebo on glycemic control, body mass index (BMI), waist circumference (WC), total daily dose (TDD) of insulin, and hypoglycemia in overweight/obese youth with T1D receiving multiple daily injections of insulin.

## Subjects and Methods

The study contract was approved by the Novo Nordisk Inc., on September 16^th^ 2010. The study protocol (S1 Protocol) was approved by the University of Massachusetts Institutional Review Board (IRB) on January 3^rd^ 2011. Study registration at ClinicalTrials.gov was begun after the IRB approval and finalized on March 21, 2011. The study’s clinical trial identification number is NCT01334125. The first study patient was recruited on March 3^rd^, 2011, after provisional approval had been granted by ClinicalTrials.gov. The study start date of February 2011 on ClinicalTrials.gov registry was not in reference to patient recruitment but to the completion of both IRB approval and IND exemption from the Food and Drug Administration. The IND reference ID is 2866975. The last study patient was recruited on August 1, 2013, and the study was closed on August 8, 2014. The authors confirm that all ongoing and related trials for this intervention are registered.

### Study Design and Setting

This study was an investigator-initiated, single-center, randomized, double-blind, parallel trial of metformin versus placebo treatments in overweight/obese youth with T1D at a university teaching hospital.

### Subjects

**Fig 1:**
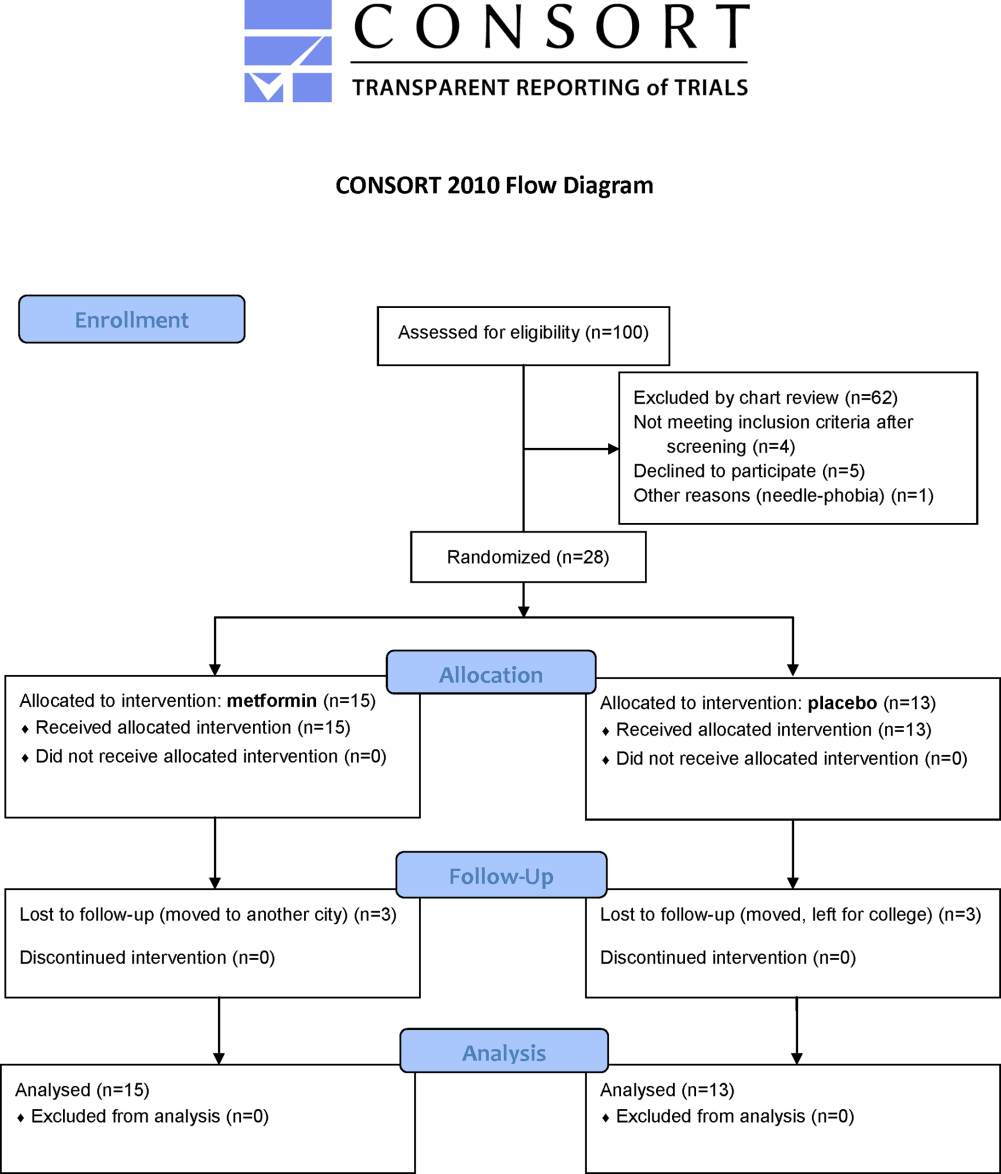
CONSORT Flow Diagram.

Written informed consent was obtained from each subject’s parent(s) and assent was also obtained from minors. Inclusion criteria were HbA1c >8% (64 mmol/mol) but <14% (130 mmol/mol), BMI >85^th^ percentile, and T1D >12-month (mo) duration. The initial age of inclusion was 10–18 years (y), however, this was later increased to 20 y to facilitate enrollment (S1 Amendment). The diagnosis of T1D was established by the presence of autoantibodies against islet antigens such as insulin, glutamic acid decarboxylase, and the protein tyrosine phosphatase-like molecule IA-2. Subjects were excluded if they were pregnant or breastfeeding; receiving weight-altering therapies; had recurrent hypoglycemia; systemic illnesses; or had a history of ≥2 episodes of diabetic ketoacidosis in the preceding 12 mo. Five subjects were excluded based on these criteria (Fig 1). All participants received once-daily subcutaneous basal insulin injections using detemir insulin; and pre-meal bolus insulin injections using insulin aspart throughout the study period. All participants were overweight or obese pubertal or post-pubertal subjects with T1D of ages 10–20 y. Fig 1 summarizes the study scheme from chart review through randomization to study conclusion.

### Methods

Participants were evaluated between 8:00–9:00 a.m. following an overnight fast.

#### Anthropometry

Anthropometric data were collected at enrollment, -3mo, 0 mo, +3mo, +6mo and at 9 mo. Height was measured to the nearest 0.1 cm using a wall-mounted stadiometer (Holtain Ltd, Crymych, Dyfed, UK). Weight was measured to the nearest 0.1 kg using an upright scale. BMI was derived using the formula weight/height^2^ (kg/m^2^), and expressed as z-scores. WC was measured to the nearest 0.1 cm at the superior border of the iliac crests.

#### Biochemical Study

Blood samples for HbA1c estimation were obtained at -3mo, 0 mo, +3mo, +6 mo and +9 mo, and were analyzed by the Umass Memorial Medical Center Biochemistry laboratory. HbA1c was measured by high pressure liquid chromatography (HPLC) which has an inter-assay variability of <1.5%, and intra-assay variability of <2.5%, and a normal range of 4.4.-6.0% [9, 10].

#### Study Supplies

Metformin and placebo were prepared as identical capsules by Boulevard Pharmaceutical Compounding Center, Worcester, Massachusetts, USA.

The study drugs (insulin detemir and insulin aspart) were shipped directly from Novo Nordisk, Inc., to the Investigational Drug Services (IDS) of the University of Massachusetts Medical School, which maintained accountability logs for receipt and dispensing of study drugs.

#### Procedure

Following enrollment, all subjects entered a run-in phase of 3-month duration during which participants were placed on insulin aspart and insulin detemir, and the treat-to-target insulin regimen (TTIR) started.

• **Methodology of Insulin Administration: Treat-to-target insulin regimen(TTIR)**

At enrollment, 23 of the 28 subjects received their basal insulin as insulin glargine, while 5 patients received their bolus insulin as insulin lispro. The 23 subjects on insulin glargine were transitioned to equivalent doses of insulin detemir; while the 5 subjects on insulin lispro were also transitioned equivalent doses of insulin aspart in a 1:1 ratio. All insulins were administered as multiple daily injections using the FlexPen insulin injecting device (FlexPen(®), Novo Nordisk A/S, Bagsvaerd, Denmark). Patients and their caregivers recorded the total and fractional daily doses of insulin in patient’s titration data sheets.

To ensure uniformity of insulin administration during the study, participants and their caregivers used a self-directed titration algorithm of (-2)-0-(+2) scale (Table 1) to adjust the dose of the subjects’ long-acting insulin, detemir, every 3rd day at bedtime to maintain fasting plasma glucose (FPG) in the normal range, as follows: increase detemir dose by 2 units if the average of the 3 prior FPG recordings is >120 mg/dL (6.7 mmol/L); reduce detemir dose by 2 units if the average of 3 FPG readings is <90 mg/dL (5.0 mmol/L), and make no change to the detemir dose if the average of 3 FPG values is between 90–120 mg/dL (5.0–6.7 mmol/L). Though the achievement of target HbA1c was encouraged during the run-in phase, it was not an inclusion/exclusion criterion for randomization.

**Table 1:** Titration Algorithm for Long-acting Insulin Analog–Detemir.

Titration Algorithm for Long-acting Insulin Analog—Detemir	
Average value of fasting plasma glucose for 3 consecutive days	Recommended long-acting insulin dose adjustments
<5.0 mmol/L (90 mg/dL)	subtract 2 units from the total dose of detemir
5.0–6.7 mmol/L (90–120 mg/dL)	no adjustments
>6.7 mmol/L (120 mg/dL)	add 2 units to the total dose of detemir

Subjects’ insulin-to-carbohydrate ratio (ICR), and the correction factor (CF) along with its component ideal blood glucose (IBG) and insulin sensitivity factor (ISF) were adjusted to maintain glycemia as shown in Table 2. Based on this titration formula, the average ICR was 1 unit of aspart per 8.0 ± 4.0 g of carbohydrate (CHO); while the average IBG used to determine the CF was 130.0 ± 10 mg/dL, and the ISF was 34.0 ± 9.0.

**Table 2:** Summary of Daily Plasma Glucose Goals.

Time	Before breakfast	Before lunch or dinner	Before bedtime	2 hours after a meal	At 3AM
Glucose level (mmol/L)	5.0–6.7	4.44–7.22	> 5.56	<12.22	>5.56
Glucose level (mg/dL)	90–120	80–130	> 100	< 220	> 100

Patients returned to the clinic 4 weeks after the initiation of TTIR and then at 12 weeks intervals till study conclusion. Follow up phone calls were made every 4 weeks to evaluate compliance and possible side effects.

• **Methodology of Glucose Data Collection**:

Fasting and non-fasting plasma glucose levels were obtained by the patient and/or caregiver by self-monitoring of patient’s capillary blood glucose using the Precision Xtra Blood Glucose Monitoring System (Abbott Diabetes Care, Alameda, CA, USA). All participants received a Study Summary Card containing a synopsis of the daily study guidelines. Parents and study subjects routinely uploaded subjects’ glucose data to the UMass MyCareTeam [11] website every 4 weeks for easy review by the study staff. Along with data recorded by families in the titration algorithm sheets, the MyCareTeam software provided data on hypoglycemic events, fasting and non-fasting plasma glucose levels, and compliance rate with capillary blood glucose monitoring.

Hypoglycemia was classified as follows: nocturnal [plasma glucose of ≤60 mg/dL (3.3 mmol/L) between 11 PM and 6 AM]; symptoms only (plasma glucose of > 60 mg/dL (3.3 mmol/L) or no measurement); minor (plasma glucose < 60 mg/dL (3.3 mmol/L); and major (hypoglycemia requiring third party assistance)[12]. To prevent nocturnal hypoglycemia, subjects were advised to maintain a pre-bedtime and nocturnal plasma glucose level of >100 mg/dL (5.6 mmol/L) (Table 2).

• **Nutrition and Exercise**:

Each participant received instructions on medical nutrition therapy from a registered dietician at the beginning of the study. No specific exercise regimen was prescribed to the subjects.

• **Randomization**:

At the conclusion of the run-in phase, subjects were randomized to either the metformin arm consisting of treatment with adjunctive metformin at a dose of 1000 mg daily for 9 months, or the control arm consisting of treatment with similar-appearing placebo capsules daily for 9 months. A metformin dose of 1000 mg was chosen because the Institutional Review Board felt that a higher dose might lead to severe hypoglycemia in patients receiving insulin therapy.

Randomization-Sequence generation: Randomization was conducted by the Investigational Drug Services (IDS) of the University of Massachusetts Medical School, using a pre-established computer-generated sequence located at www.randomization.com. Randomization protocol was 1:1 (metformin: placebo) and was blocked for every 10 subjects.

Randomization-Allocation concealment: Double-blinded treatments were allocated using sequentially-numbered drug containers. Concealed treatment allocation was made by IDS, which secured blinding codes during the trial. IDS maintained a sealed copy of the randomization sequence at the investigation site in case of need for emergency unblinding.

Randomization-Implementation: The IDS established the randomization sequence. The trial endocrinologists enrolled patients to the study. A pharmacist, unconnected with the study, assigned participants to the groups.

Blinding: Patients and investigators were blinded to the identity of the study medication and placebo. IDS maintained blinding information throughout the study duration. At study conclusion, the randomization code was decrypted in a two-step procedure as follows: first step: treatment A or B; second step: A = metformin and B = placebo. All statistical analyses were performed after the second step of unblinding.

#### Objectives

The objective of the trial was to determine the safety and efficacy of adjunctive metformin therapy compared to placebo during a 9-mo treatment period in overweight/obese youth with T1D and poor glycemic control.

#### Outcomes

Primary outcome: Comparison of the baseline-adjusted differences in HbA1c between the metformin and placebo groups during the trial.

Secondary outcomes: Pre-specified secondary outcomes included additional parameters that are associated with glycemic control such as total daily dose (TDD) of insulin, TDD of short-acting insulin, TDD of long-acting insulin, fasting plasma glucose, and hypoglycemia assessed at baseline and every 3 months till study completion. Anthropometric parameters including body mass index, and waist circumference were assessed. Safety variables included pregnancy tests in female subjects of child-bearing age, liver function tests, and creatinine.

Ancillary outcomes: Changes in the correction factor (CF), insulin to carbohydrate ratio (ICR), insulin sensitivity factor (ISF), ideal blood glucose (IBG) during the trial, and the change in HbA1c during the run-in period.

#### Protocol Deviations

Treatment was withheld for 2 days in a subject during hospital admission for the management of severe hypoglycemia; and for 3 days in another patient during an admission for acute appendicitis necessitating appendectomy; and in two patients for 2 days each during hospital admissions for the management of diabetic ketoacidosis. All these subjects were later determined to be in the metformin group. This trial did not enroll the stipulated sample size as the study was stopped before reaching enrollment target because most youth were reluctant to commit to this long-term study (S1 CONSORT Checklist).

### Statistical Analyses

The power calculation for this study was done for a repeated-measures model using GPower (v.3.1.6, Universität Kiel, Germany). We based this calculation on the level of HbA1c, because it required the smallest effect difference for a biologically meaningful comparison. We used a confidence level of 95% (Z _(1-α/2)_ = 1.96 for a two-tailed test) and a statistical power of 80% (Z _(1-β)_ = 0.84). Our assumptions were that the use of metformin would reduce average HbA1c values over time by 0.75%, and that the standard deviation for these estimates would be ±1.25. We also assumed a within subject correlation of 0.500. Our calculated group sizes of 29 individuals for each arm of the trial yielded an actual power of 81.1%.

Subject characteristics were summarized using means and standard deviations (SD). Group-specific comparisons of anthropometric and biochemical parameters were performed at baseline using paired *t* tests or its non-parametric equivalent where indicated. Where doubts arose about the Normality of our variables, we carried out the above analyses on log-transformed values. Independent proportions (e.g., race, gender) were compared using Fisher’s exact test. Differences in treatment effects between the randomized groups were evaluated by comparison of the three study time points (+3mo, +6mo, +9mo), for each of the outcomes of interest: HbA1c values, fasting blood glucose, total daily dose of insulin, and fractional daily dose of short- and long-acting insulin. Using the General Linear Model (GLM) procedure, individual two-way mixed ANOVA models were fitted for each of our outcomes of interest, with treatment type (metformin or placebo) as the fixed effect and baseline values as covariates.

We had three missing HbA1c measurements in our dataset. Rather than allowing case deletion, which would have further reduced our power, we imputed the missing values by averaging the HbA1c measurements immediately before and after the missing datapoints. For this, we assumed these data were missing completely at random, and considered that our very small fraction of missing values (0.03%) would not introduce bias. The correlation structure used was first order autoregressive structure with homogenous variance (ARI). Analyses were based on the intent-to-treat principle and were performed using SPSS v.22 (IBM Corporation, Armonk, NY).

## Results

### Baseline Characteristics

Table 3 shows a comparison of the baseline/randomization characteristics of the metformin (n = 15) and the placebo (n = 13) groups. The difference in the absolute TDD of insulin per kg body weight/day appears to be principally due to a higher fractional dose of fast-acting insulin in the placebo group.

**Table 3:** Anthropometric and Biochemical Characteristics of the Subjects and Controls at Baseline/Randomization. Table 3 shows the group-specific comparisons of anthropometric and biochemical parameters at baseline using paired *t* tests or its non-parametric equivalent where indicated. Subject characteristics were summarized using means ± standard deviations (SD). Independent proportions (e.g., race, gender) were compared using Fisher’s exact test*. SDS standard deviation score; WC waist circumference; TDD total daily dose; HbA1c hemoglobin A1c. All participants received long-acting insulin detemir and short-acting insulin aspart. Significant *p* values are bolded.

Parameters	Metformin (n = 15)	Placebo (n = 13)	*p*
Age	15.0 ± 2.5	14.5 ± 3.1	0.650
Gender (males)	8/15 (53.3%)	5/13 (38.5%)	0.343*
Ethnicity (Caucasian+Hispanics)	9/15 (60.0%)	11/13 (84.6%)	0.155*
Pubertal (Tanner II-IV)	8/15 (53.3%)	5/13 (38.5%)	0.343*
Height	162.4 ± 11.0	159.1 ± 13.9	0.491
Height SDS	-0.1 ± 1.2	0.23 ± 1.1	0.450
Weight	75.5 ± 25.0	70.8 ± 17.9	0.579
Weight SDS	1.5 ± 0.9	1.7 ± 0.5	0.528
BMI	28.2 ± 6.6	27.5 ± 3.7	0.741
BMI SDS	1.6 ± 0.6	1.7 ± 0.3	0.546
WC (cm)	89.7 ± 17.0	97.5 ± 22.7	0.318
Systolic Blood pressure (mmHg)	115.1 ± 18.9	117.5 ± 19.4	0.743
Diastolic Blood pressure (mmHg)	78.5 ± 10.7	78.5 ± 11.9	0.987
Fasting Plasma Glucose (mmol/L)	10.7 ± 3.0	10.8 ± 3.6	0.973
Fasting Plasma Glucose (mg/dL)	193.1 ± 54.1	193.9 ± 64.5	0.973
HbA1c (%)	9.3 ± 1.5	8.7 ± 0.4	0.177
HbA1c (mmol/mol)	77.9 ± 16.5	71.2 ± 4.8	0.177
Duration of disease (years)	5.7 ± 4.4	5.7 ± 5.0	0.991
TDD insulin (units)	84.0 ± 42.9	105.7 ± 43.9	0.198
TDD insulin (units/kg/day)	1.1 ± 0.2	1.44 ± 0.5	**0.018**
TDD short-acting insulin only (units)	36.7 ± 22.8	49.5 ± 25.8	0.175
TDD short-acting insulin per kg body weight per day (units/kg/day)	0.5 ± 0.2	0.7 ± 0.4	0.067
TDD long-acting insulin only (units)	47.2 ± 23.2	56.2 ± 27.0	0.350
TDD long-acting insulin per kg body weight per day (units/kg/day)	0.6 ± 0.1	0.8 ± 0.3	0.084

### Changes in anthropometric parameters

**Fig 2:**
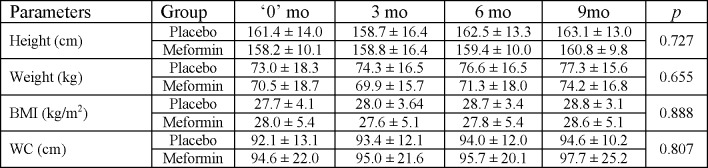
Comparison of Anthropometric Parameters during the Trial. This figure shows the changes in anthropometric characteristics between the groups during the trial. Analysis was performed by repeated measures ANOVA by comparing the 4 study time points (baseline, +3mo, +6mo, +9mo) for each anthropometric parameter.

There were no significant differences between the placebo (n = 13) and metformin (n = 15) groups from baseline to study conclusion for the following parameters: systolic blood pressure (BP) (p = 0.524), diastolic BP (p = 0.170), weight SDS, WC, and BMI SDS (Fig 2).

### Changes in hemoglobin A1c

**Fig 3:**
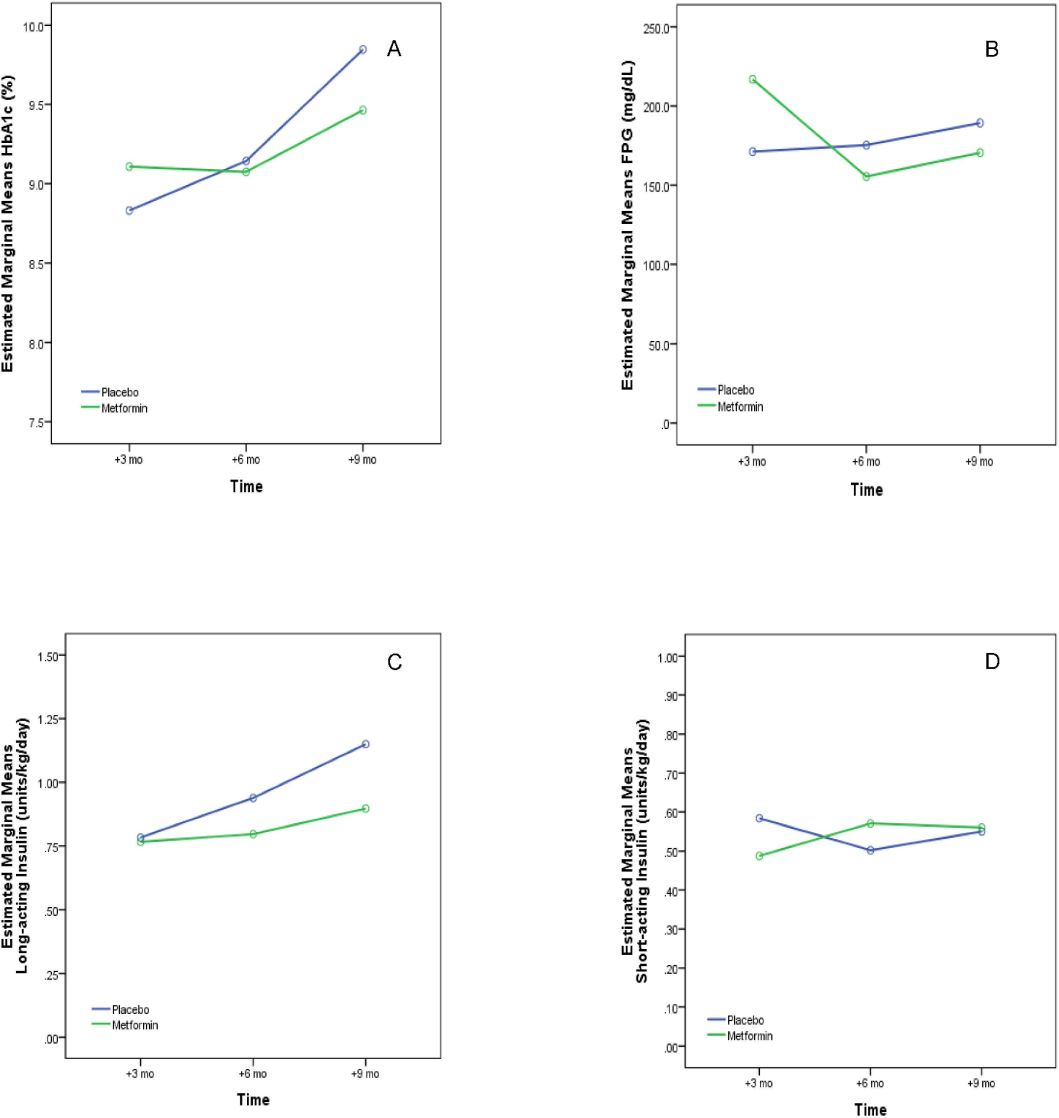
Estimated marginal means for changes in outcome parameters A, B, C, D. A. Hemoglobin A1c: Over the course of our 9 months of observation, the between-treatment differences in HbA1c of 0.4% (9.85% [8.82 to 10.88] for placebo versus 9.46% [8.47 to 10.46] for metformin) was not significant (p = 0.903). B. Fasting plasma glucose (FPG): For the duration of the interventional phase of the study, the 18.9 mg/dL difference in FPG between the groups was not significant (189.4 mg/dL [133.2 to 245.6] for placebo versus 170.5 mg/dL [114.3 to 226.7] for metformin), (p = 0.927). C. Total daily dose (TDD) of long-acting insulin: the 0.25 unit/ kg TDD decrease of long-acting insulin per kg body weight per day (1.15 units/kg [0.89 to 1.41] for placebo versus 0.90 units/kg [0.64 to 1.16] for metformin) was not significant (p = 0.221). D. Total daily dose of short-acting insulin: the 0.01 unit/kg/day for the TDD of short-acting insulin per kg body weight/day did not vary between the groups (p = 0.936). The difference between the time points was marginally significant (p = 0.090), as well as the interaction between time and groups (p = 0.079).

**Fig 4:**
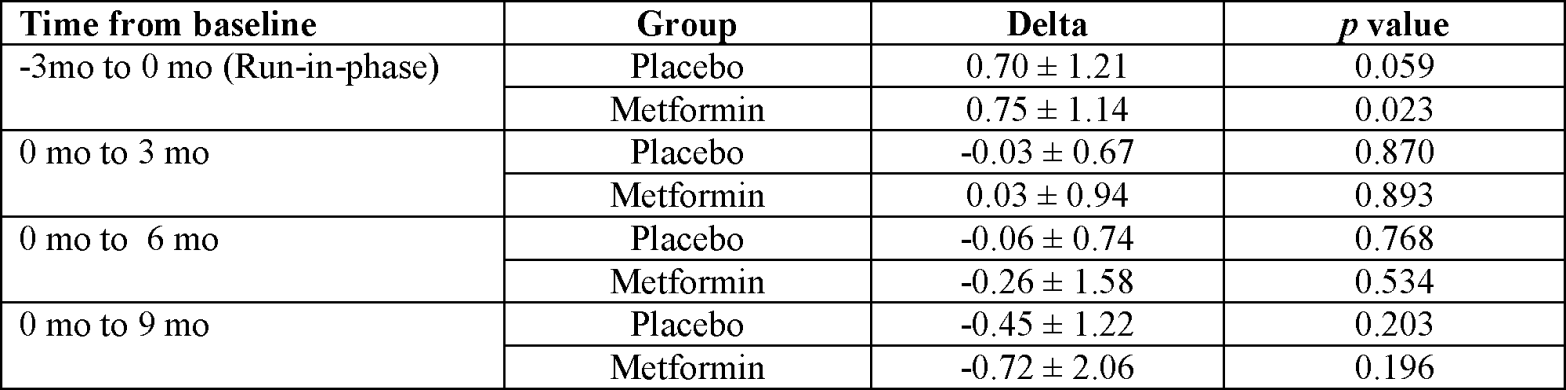
Comparison of changes in hemoglobin A1c values between the placebo and metformin groups during the trial.

Over the course of our 9 months of observation, the between-treatment differences in HbA1c of 0.39% (9.85% [8.82 to 10.88] for placebo versus 9.46% [8.47 to 10.46] for metformin) was not significant (p = 0.903) (Fig 3A). In this model, the assumption of sphericity was not fulfilled (Mauchly’s test p<0.001) so we used the Greenhouse-Geisser correction for our degrees of freedom. There was no interaction between time and group (p = 0.290). There were no significant changes in A1c in both the metformin and placebo groups at each study time point compared to baseline A1c (Fig 4).

### Changes in Fasting Plasma Glucose (FPG)

FPG decreased significantly in subjects who were later randomized to the metformin group from -3mo to 0 mo during the run-in phase (p = 0.027), but there was no significant change in FPG in the subjects who were later randomized to the placebo group during the run in phase (p = 0.127) (Fig 2B).

For the duration of the interventional phase of the study, the 18.9 mg/dL difference in FPG between the groups was not significant (189.4 mg/dL [133.2 to 245.6] for placebo versus 170.5 mg/dL [114.3 to 226.7] for metformin), (p = 0.927). No significant interaction was found between time and group (p = 0.089).

### Changes in Total Daily Dose of Insulin

The change in absolute total daily dose (TDD) of insulin per kg body weight per day of 0.31 units/kg (1.73 units/kg [1.44 to 2.02] for placebo versus 1.42 units/kg [1.13 to 1.71] for metformin) did not increase significantly over time (p = 0.245), with no interaction between time and group (p = 0.362).

Similarly, the 0.25 unit/kg TDD decrease of long-acting insulin per kg body weight per day (1.15 units/kg [0.89 to 1.41] for placebo versus 0.90 units/kg [0.64 to 1.16] for metformin) was not significant (p = 0.221). Neither was the interaction between time and group (p = 0.269) (Fig 2C).

The 0.01 unit/kg change in the TDD of short-acting insulin per kg body weight did not vary between the groups (p = 0.936) (Fig 2D). The difference between the time points was marginally significant (p = 0.090), as well as the interaction between time and groups (p = 0.079).

### Changes in the Correction Factor, Insulin to carbohydrate ratio (ICR), Insulin sensitivity factor (ISF), Ideal Blood glucose (IBG)

A comparison of the changes in insulin-to-carbohydrate ratio (ICR), ideal blood glucose (IBG), and insulin sensitivity factor (ISF) from baseline at randomization to the end of the study showed no significant difference in ICR in the metformin group: 1 unit aspart per 8.5 ± 4.0 g of carbohydrate vs. 8.6 ± 4.0, (p = 0.91); or the placebo group 8.15 ± 5.3 vs. 7.62 ± 5.42, (p = 0.304).

There were equally no differences between baseline and end of study values for ISF in the placebo group 35.4 ± 13.3 vs. 34.6 ± 12.8, (p = 0.687); or in the metformin group 38.3 ± 13.5 vs. 33.0 ± 10.0, (p = 0.100). Furthermore, the IBG was similar at baseline and at the end of study in the placebo group: 128.9 ± 11.9 mg/dL vs. 129.2 ± 12.1, (p = 0.819); and also in the metformin group: 133.0 ± 14.6 vs. 133.0 ± 11.9, (p = 1.00).

A repeated measures ANOVA showed no differences in ISF (p = 0.879), IBG (p = 0.383), and ICR (p = 0.696) between the placebo and metformin group from baseline to the conclusion of the study.

### Occurrence of Hypoglycemia

Minor hypoglycemia was reported in 3 subjects (20.0%) in the metformin arm, and 2 subjects (15.4%) in the placebo arm (p = 1.00). Nocturnal hypoglycemia was reported in 2 subjects (13.3%) in the metformin group, and 2 subjects (15.4%) in the placebo group (p = 1.00). One instance of major hypoglycemic event occurred in a subject in the metformin arm following a period of reduced caloric intake. The patient recovered fully without sequalae. No instance of major hypoglycemia was reported in the placebo arm.

### Occurrence of Other Adverse Events

Among the subjects in the metformin arm, there was one subject with acute appendicitis necessitating appendectomy, and single episodes of eye infection that resolved on antibiotic therapy, a case of mild transient microalbuminuria, and two instances of diabetic ketoacidosis that occurred during intercurrent illnesses. Among the subjects in the placebo arm, there were single episodes of upper respiratory tract infection, pneumonia, otitis media, and hidradenitis. All subjects recovered from the adverse effects without sequalae.

### Compliance

Compliance was monitored during the study by the frequent review of MyCareTeam software downloads, counting of pills in the dosettes during clinic visits, and review of subjects’ home documentation of blood glucose data. Analysis of compliance parameters and pharmacy records showed no difference in compliance between the two groups. Compliance rate, which was initially at 90% for both arms during the run-in phase, decreased to 65% at the interval between the 6^th^ and 9^th^ month visit.

## Discussion

In this nine-month randomized, double blind, placebo controlled trial, we report a 0.4% lower HbA1c value in the metformin group compared to the placebo group. This glycemic finding was associated with non-significant decreases in FPG, and TDD of long-acting insulin compared to placebo. This clinically significant difference [13] in HbA1c may not have reached statistical significance due to type 2 error arising from inadequate sample size.

To our knowledge, this is the longest randomized, double-blind, placebo controlled trial of adjunctive metformin therapy in overweight/obese youth of both genders with T1D. This study is further distinguished by its employment of a standardized insulin delivery protocol, the treat-to-target insulin regimen, to ensure uniformity of insulin titration in both arms of the study; the utilization of MyCareTeam software to capture glucose data for glycemic monitoring and analysis; and the use of uniform insulin analogs delivered via multiple daily insulin injections only.

Published reports on adjunctive metformin therapy in overweight and obese patients with T1D have reached differing conclusions [1, 2, 6, 14–18]. A recent meta-analysis of 197 studies on the use of adjunctive metformin therapy in children and adults with T1D concluded that metformin therapy reduces insulin dose requirements in T1D but not HbA1c value[16]. A recent 9-mo RCT that examined the effect of metformin for the treatment of hyperandrogenism in 24 female adolescents and young women with T1D, included glycemic endpoints in their analysis and found no differences in HbA1c and total daily doses of insulin between the placebo and metformin groups[19]. This study differed from our trial in that the investigators enrolled only female subjects; used only 850 mg of metformin daily in the treatment arm; and recruited both normal-weight and overweight/obese subjects. They neither evaluated changes in fasting glucose levels nor employed a standardized insulin treatment protocol to ensure uniformity of glycemic control in both the placebo and metformin groups. They also did not investigate the changes in the doses of fast-acting- and long acting insulins in both groups. Another trial, a 6-month RCT of adjunctive metformin in 74 youth with T1D concluded that metformin therapy reduces insulin dose requirements in T1D but not HbA1c value[1].This study, though adequately powered, was limited by its relatively short duration.

In contrast, some studies in youth have reported significant reduction in HbA1c in those who received adjunctive metformin therapy [2, 6, 17, 18]. These studies were, however, limited by their short duration of 3 months[2, 6], open label design and very small sample size of 9 subjects [17] and 10 subjects[18]. Moreover, the two RCTs that reported reductions in HbA1c [2, 6] did not categorically recruit overweight/obese children and adolescents with T1D, and did not employ a titration regimen to ensure uniformity of insulin dosing and glycemic control.

Taken together, despite the lack of consensus on the efficacy of adjunctive metformin on HbA1c in T1D, our study suggests that prolonged treatment with adjunctive metformin could result in a clinically significant[13] reduction in HbA1c in overweight/obese children and adolescents with T1D.

The elevated mean HbA1c values in our study are similar to the findings from a recent national report on glycemic control in children and adolescents with T1D which showed higher mean HbA1c level [8.4 ± 1.4% for whites, 9.6 ± 1.9% for blacks, and 8.7 ± 1.6% for Hispanics] than the recently recommended HbA1c target of <7.5% by the American Diabetes Association[20]. Furthermore, our finding of suboptimal glycemic control despite increasing insulin dose in the placebo group is consistent with the results of the Diabetes Control and Complications Trial (DCCT) [21], which reported that the average of HbA1c values in adolescents with T1D was 1% higher than in adults in both the conventional and intensive treatment groups, despite receiving more insulin; and attempts to increase insulin doses led to weight gain and worsening of glycemic control. The proposed mechanism for this deterioration in glycemic control was termed insulin resistance of puberty, which is believed to be driven by the significantly elevated levels of anti-insulin hormones namely growth hormone and sex steroids, during the period of active pubertal maturation.

Apart from IR of puberty, other factors may have contributed to the failure to achieve optimal glycemia in this cohort. Differences in subjects’ adherence to insulin therapy may have led to poor glycemic outcome. Equally, some patients could have adjusted their insulin doses to maintain glycemic targets that were substantially higher than those directed by the investigators. It could also be due to a combination of these two factors.

The significant reduction in insulin requirement in our metformin cohort is consistent with improved insulin sensitivity [22]. The lack of a significant change in ISF in the metformin group suggests that the mechanism for this reduction in insulin requirement was principally by increasing hepatic insulin sensitivity through the inhibition of hepatic gluconeogenesis and consequent reduction in hepatic glucose production [2]. However, though the lack of a statistically-significant reduction in HbA1c suggests that insulin resistance may not be the sole mechanism for poor glycemic control in overweight/obese youth with T1D, our small sample size may have introduced type 2 statistical errors that prevented the detection of significant differences in HbA1c between the groups.

Several socio-demographic and therapeutic factors such as race/ethnicity, socioeconomic status, parental education, parental involvement in diabetes management, family dynamics, and whether a patient receives care from an endocrinologist[23] have been associated with poor glycemic control in youth [23–25]. These variables were considered in our analysis as all of our subjects received their care from pediatric endocrinologists, had parental involvement in their diabetes care, were of low to medium socio-economic status, and mostly of white or Hispanic ethnic groups.

This RCT has a number of limitations that should be taken into account in the interpretation of the results. The small sample size is a major limitation and this could have limited our ability to detect significant differences in the outcome parameters. Such type 2 errors might indicate that we had insufficient power to reject the null hypothesis despite the fact that this hypothesis is false. This is particularly important in interpreting the results that were nearly significant such as the change in the TDD of long-acting insulin in which the metformin group had a consistently lower requirement for long-acting insulin throughout the trial compared to the placebo group (p = 0.221). It is possible that a significant difference could have been detected with an adequate sample size.

Secondly, though the significantly reduced insulin requirement in the metformin group was consistent with increased insulin sensitivity in that cohort, no formal measurement of insulin resistance using techniques such as hyperinsulinemic euglycemic clamp was conducted. Furthermore, changes in physical activities during the study, which could alter insulin sensitivity, were not measured; and plasma or urine metformin levels were not obtained to monitor adherence. Intake of calories and macronutrients was not monitored and analyzed as these could represent strong modifiers of glycemic control in patients with T1D.

The trial was stopped before reaching enrollment target because most youth were reluctant to commit to this long-term study. An analysis of the characteristics of the 10 subjects who were consented for the study but not randomized, showed no differences in anthropometric and glycemic characteristics between the randomized subjects and the 6 dropouts. The remaining 4 subjects were excluded because they either had an HbA1c value of <8% or BMI of <85% on the day of their scheduled randomization.

The study’s strengths lie in its randomized controlled design, the long duration of intervention, the enrollment criteria, and the use of a common insulin treatment protocol of multiple daily insulin injections only, thus ensuring a uniformity of timing and delivery of both short- and long-acting insulins. This is the first trial to employ a standardized insulin titration protocol, the treat-to-target insulin regimen, in the investigation of the role of adjunctive metformin therapy in youth with T1D. The employment of treat-to-target insulin regimen ensured that all participants used the same algorithm for insulin dose adjustment throughout the duration of the study. This protocol addressed the problem of the uniformity of insulin administration, which has been a recurrent confounder in other trials on adjunctive metformin therapy in T1D. Further strengths of the study include the use of a common insulin analog for the short-acting and long-acting insulin formulations; the use of a single basal insulin dose given every evening to ensure accuracy of titration of long-acting insulin dose to FPG levels, and the incorporation of the MyCareTeam software to capture glucose readings for the entire duration of the study. The trial’s retention rate of 78.6% ensured that the completers were representative of the original cohort. This is the first trial in youth to accurately determine that the differential increase in insulin requirement in the placebo group was a function of escalations in the dose of long-acting insulin, and not the short-acting insulin dose. Thus, the long duration of this trial ensured that our conclusions were free from initial Hawthorne effects, and the institution of a standardized insulin delivery protocol allowed conclusions to be drawn on the effect of adjunctive metformin therapy on glycemic control and insulin requirements that were not possible in previous studies in this field. Therefore, though this trial’s relatively small sample size limits the generalizability of these findings, the study’s randomized, placebo-controlled design and the novel measures implemented to limit confounding variables, ensured the validity of these results.

## Conclusions

In this nine-month randomized, double blind, placebo controlled trial, we report a 0.4% lower HbA1c value in the metformin group compared to the placebo group. This clinically significant difference [13] in HbA1c did not reach statistical significance most likely due to type 2 error arising from inadequate sample size. This reduction in HbA1c suggests that adiposity-associated insulin resistance may be one of the primary mechanisms for poor glycemic control in these patients while other factors such as socio-demographic, hormonal, and therapeutic factors may play a secondary role on glycemic control in this population.

## Supporting Information

S1 CONSORT Checklist (DOC)

S1 Protocol (DOC)

S1 Amendment (PDF)
