# Supplementary material for: A Randomized, Double-Blind, Placebo-Controlled Trial of Adjunctive Metformin Therapy in Overweight/Obese Youth with Type 1 Diabetes
Source: PLoS One. 2015 Sep 14;10(9):e0137525. doi: 10.1371/journal.pone.0137525 (PMC4569440; doi:10.1371/journal.pone.0137525)
Supplement: S1 Protocol — (DOC) [file pone.0137525.s002.doc]

**APPLICATION FOR APPROVAL OF UMMS HUMAN STUDIES**

UNIVERSITY OF MASSACHUSETTS MEDICAL SCHOOL

UMass Memorial Medical Center

NOTICE TO INVESTIGATOR

**THE PRINCIPAL INVESTIGATOR IS RESPONSIBLE FOR THE CONTENT OF THIS APPLICATION AND *MUST* PROOF READ THE FINAL VERSION OF THE APPLICATION FORM BEFORE IT IS SUBMITTED.** Errors in the application reflect poorly on the PI’s oversight of the research. **FAILURE TO ADEQUATELY REVIEW THE APPLICATION WILL PUT THE STUDY AT HIGH RISK OF BEING TABLED UNTIL THE NEXT MEETING.**

Before the IRB meeting deadline, you must submit **ONE COPY** of the completed IRB application, including all 7 of the sections. All signatures and attachments **MUST** be in place. **The application and consent form must be numbered.** This packet will be pre-reviewed in the Human Subjects Office and returned to you. Copies should not be made until the pre-review is completed.

This administrative review is done to prevent receiving applications that are poorly prepared and unacceptable to the Committee. You are urged to prepare this application and consent form carefully. The two Human Subjects Committees review 10-20 protocols a month. The Committees are composed of individuals who donate a considerable amount of their time to this effort, and your careful attention to accurate, complete information and grammar are fully anticipated. The Human Subjects staff and the IRB reviewers will return the submission to you if this is not the case, resulting in an unnecessary delay in study review and your anticipated study initiation

**INSTRUCTIONS**

**RADIATION OR DNA/CELL LINES**

If the subjects receive any radiation, please contact the Radiation Safety Committee (RSC) at 508-856-3208 to discuss the possibility of RSC review. If RSC review is needed, IRB approval will not be given until RSC approval has been obtained.

If the subjects receive any rDNA vaccines, retroviruses, adenovirus derivative vectors, or autologous modified tissue, please contact the Institutional Biosafety Committee (IBC) at 508-856-5416 to discuss the possibility of IBC review and registration. If laboratory personnel will be involved with rDNA vaccines, retroviruses, adenovirus derivative vectors, or autologous modified tissue this must also be registered with the IBC. If review is needed, IRB approval will not be given until IBC approval has been obtained.

**SIGNATURES**

1. The PI signs Section III and Section VII the Informational Drug Data Form (IDDF).

2. The Chair and the Chief of the PI’s Department/Division sign Section IV.

3. Additional signatures may also be required in section IV, please review.

3. All Research Personnel, including the PI, sign Section VI.

**PROCEDURES FOR SUBMITTING A COMPLETED APPLICATION**

If you are unsure about the type of review required by your study, or are inexperienced in completing IRB applications, it is strongly recommended that you provide a copy of a reasonably complete draft version. A substantive preliminary review by the Human Subjects Office will be performed. This preliminary review gives you the opportunity to address issues before the meeting and will save you time in the long run. Obviously, this review must be done well in advance of the IRB meeting deadline.

**EXPEDITED REVIEW**

If the study qualifies for Expedited Review (determined by the Human Subjects Office after review) the original and three copies of the final version of the application will be required. Two Committee members will review the protocol. This process usually takes approximately three weeks.

**FULL COMMITTEE REVIEW**

If the full Committee must review the study, the original and twenty copies will be needed (one for each member of the Committee). **Please note that the original copy of the full application and consent form must be sent to the Human Subjects Office for initial administrative review before the Principal Investigator makes twenty copies for the Committee.**

Meetings are scheduled for the first and third Tuesday of each month at 4:00 P.M. (except for the months of July and August when the Committee meets once each month). [Meeting dates and deadline dates](http://www.umassmed.edu/subjects/human/meetings.cfm) are available on our [web page](http://www.umassmed.edu/subjects/human/) and are subject to change.

Each protocol is reviewed by two committee members prior to the meeting, and the investigator may be contacted to respond to concerns. You will be notified of the date and location of the meeting. Most Principal Investigators do not have to attend the meeting, but you are asked to be on call via your pager or telephone between the meeting hours of 4-6 p.m.

**AMENDMENTS**

The Human Subjects Office or the IRB must review any amendment or change in a protocol or consent form. No changes may be instituted until the investigator has received written approval of the revision from the Committee.

**YEARLY REVIEW AND REAPPROVAL**

Approved studies must receive re-approval at least once a year and more often if required by the Committee. A notice will be sent to you before the re-approval is due; approximately 2 months prior to the expiration date. Re-approval must occur within 30 days of the expiration date and appropriate planning must take place to meet this required deadline.

Please contact the Human Subjects Office at 856-4261 if you need additional information.

**CONTENTS OF THIS APPLICATION**

I PRINCIPAL INVESTIGATORS CHECK LIST

II PROTOCOL SUMMARY SHEET

III PRINCIPAL INVESTIGATORS ASSURANCE

IV DEPARTMENTAL APPROVAL

V DESCRIPTION OF RESEARCH PROJECT

VI CERTIFICATION OF APPROVAL

VII INFORMATIONAL DRUG DATA FORM

**SECTION I**

|  | |
| --- | --- |
| Before this application is submitted to the Research Subjects Office, the following must be done. | |
| Please indicate by stating “YES” OR “N/A” (not applicable) that you have reviewed the packet and | |
| Have accomplished these tasks as they apply to your study. | |
|  | |
| **IN THE APPLICATION SECTION** | |
|  | |
| Yes | Completed Protocol Summary Sheet Section II |
|  |  |
| Yes | Completed & obtained signatures on the P.I.’s Assurance Section III and |
|  | Obtained signed agreement forms from all cooperating faculty Section V and departments Section VII. |
|  |  |
| N/A | Obtained approval from Radiation Safety Committee or submitted protocol to the RSC |
|  |  |
| N/A | Provided the Investigational New Drug (IND #) on the Protocol Summary Sheet Section II |
|  |  |
| YES | If the study is grant funded, please contact the Research Funding Office at 508-856-2119 for more information. If the study is industry supported, please contact Office of Clinical Research at 508-856-5015 |
|  |  |
| YES | Provided 1 copy of the Sponsor Protocol or the “body” of the research grant (e.g. sections a through e of the |
|  | Research Plan of an NIH grant). HUMAN SUBJECTS USE MUST BE IDENTICAL IN GRANT/COMPANY PROTOCOL |
|  | AND IRB APPLICATION. |
|  |  |
| N/A | Provided 1 copy of the Investigator’s Drug Brochure |
|  |  |
| YES | Numbered the pages of the Protocol body. |
|  |  |
| N/A | Obtained approval from the Institutional Biosafety Committee (IBC) or submitted the protocol to the IBC Committee for review. |
|  | **IN THE CONSENT FORM** |
|  |  |
| YES | Indicated that subjects will sign a written consent form. |
|  |  |
| YES | Provided a consent form in standard UMMS format |
|  |  |
| YES | Wrote the consent form in the second person and at a 7th grade level. |
|  |  |
| YES | Numbered the pages of the consent form appropriately. (e.g. Page 1 of 4, Page 2 of 4) |
|  |  |
| YES | Indicated that verbal consent will be obtained if written consent is not being obtained. |
|  |  |
| N/A | Provided a fact sheet for the patient. (A fact sheet should be included for complex, lengthy, or high risk studies.) |
|  |  |

**SECTION II**

**PROTOCOL SUMMARY SHEET**

| Today's Date: | | | | | 1-14-2011 | | | | | |  | | | | | | | | | | | | | | | | | | | | |
| --- | --- | --- | --- | --- | --- | --- | --- | --- | --- | --- | --- | --- | --- | --- | --- | --- | --- | --- | --- | --- | --- | --- | --- | --- | --- | --- | --- | --- | --- | --- | --- |
| P.I. Name: | | | **BENJAMIN U. NWOSU** | | | | | | | | | | | | | | Degree: | | | **MD** | | | | | | | | |  | | |
| (PI Must be UMMS Faculty Member) | | | | | | | | | | | | | | | | | Faculty Title: | | | | Assistant Professor | | | | | | | | | | |
| Department: | | | | PEDIATRICS | | | | | | | | | | | | | | | | | | | | | | | | | | | |
| Division Name: | | | | ENDOCRINOLOGY | | | | | | | | | | | | | Duration of the Study: | | | | | | | | | 24 months | | | | | |
| Phone # | | 508-334-7872 | | | | | | | | | |  | | | | | Total # of subjects at UMMHC: | | | | | | | | | | | 200 | | | |
| Beeper/Pager#: | | | | | 508-426-3499 | | | | | | |  | | | | | | | | | | | | | | | | | | | |
| Email Address: | | | | Benjamin.[nwosu@umassmemorial.org](mailto:nwosu@umassmemorial.org) | | | | | | | | | | | | | | | | | | | | | | | | | | | |
| Title of Study: | | | | | | | Glycemic Control in Children and Adolescents with Double Diabetes: Trial of Optimized Insulin-Metformin Regimen  **Protocol Number: 13938** | | | | | | | | | | | | | | | | | | | | | | | |  |
| (Include Protocol #) | | | | | | |  |  |  |  |  |  |  |  |  |  |  |  |  |  |  |  |  |  |  |  |  |  |  |  |  |
|  |  |  |  |  |  |  |  |  |  |  |  |  |  |  |  |  |  |  |  |  |  |  |  |  |  |  |  |  |  |  |  |
|  |  |  |  |  |  |  |  |  |  |  |  |  |  |  |  |  |  |  |  |  |  |  |  |  |  |  |  |  |  |  |  |
|  |  |  |  |  |  |  |  |  |  |  |  |  |  |  |  |  |  |  |  |  |  |  |  |  |  |  |  |  |  |  |  |
|  |  |  |  |  |  |  |  |  |  |  |  |  |  |  |  |  |  |  |  |  |  |  |  |  |  |  |  |  |  |  |  |
|  | | | | | | | | | | | | | | | | | | | **"X" below which sites will participate** | | | | | | | | | | | |  |
| Contact Person Name | | | | | | | | Benjamin U. Nwosu | | | | | Phone # | 508-334-7872 | | | | | University: | | | | X | | | | | | | |  |
| Pager # | 3499 | | | | | | | | |  | | | | | | | | | Memorial : | | | |  | | | | | | | |  |
| Identify Condition being studied: | | | | | | | | | | Double Diabetes | | | | | | | | | Marlborough: | | | | |  | | | | | | |  |
|  | | | | | | | | | | | | | | | | | | | Shriver Center: | | | | | |  | | | | | |  |
| Source of Funding: | | | | | | Novo Nordisk, Inc. | | | | | | | | | | | | | Others: | | |  | | | | | | | | |  |
| **Protocol #** | | | | | | | | | | | | | | | | | | | | | | | | | | | | | | | |
| **DEVICE INFORMATION** | | | | | | | | | | | | | | | | | |  | | | | | | | | **DRUG INFORMATION** | | | | | |
| Please provide IDE# if not approved by FDA | | | | | | | | | | | | | | |  | | | In the table below, list all drugs being used. If the drug is considered investigational by the FDA you must include the IND# assigned by the FDA. Please "X” approved or investigational. | | | | | | | | | | | | | |
| Device Name | | | | | | Approved | | | Investigational | | | | IDE# | |  |  |  |  |  |  |  |  |  |  |  |  |  |  |  |  |  |
|  |  |  |  |  |  |  |  |  |  |  |  |  |  |  |  |  |  |  |  |  |  |  |  |  |  |  |  |  |  |  |  |
|  |  |  |  |  |  |  |  |  |  |  |  |  |  |  |  |  |  |  |  |  |  |  |  |  |  |  |  |  |  |  |  |
|  |  |  |  |  |  |  |  |  |  |  |  |  |  |  |  |  |  |  |  |  |  |  |  |  |  |  |  |  |  |  |  |
|  | | | | | | | | | | | | | | | | | | | | | | | | | | | | | | | |
| **USE SPACE BELOW FOR COMMENTS OR ADDITIONAL DRUG INFORMATION** | | | | | | | | | | | | | | | | Drug Name: | | | | | | | | | Approved | | Inves. | | | IND# | |
| The Food and Drug Administration has reviewed this study and granted us an IND exemption for these drugs. The reference ID is 2866975. | | | | | | | | | | | | | | | | Metformin | | | | | | | | | X | |  | | |  | |
|  |  |  |  |  |  |  |  |  |  |  |  |  |  |  |  | Insulin aspart | | | | | | | | | X | |  | | |  | |
|  |  |  |  |  |  |  |  |  |  |  |  |  |  |  |  | Insulin detemir | | | | | | | | | X | |  | | |  | |
|  |  |  |  |  |  |  |  |  |  |  |  |  |  |  |  |  |  |  |  |  |  |  |  |  |  |  |  |  |  |  |  |
|  |  |  |  |  |  |  |  |  |  |  |  |  |  |  |  |  |  |  |  |  |  |  |  |  |  |  |  |  |  |  |  |
|  |  |  |  |  |  |  |  |  |  |  |  |  |  |  |  |  |  |  |  |  |  |  |  |  |  |  |  |  |  |  |  |

**DECRIBE THE RESEARCH BY CHECKING ALL THE ITEMS “YES” OR “NO”**

| Yes | No |  | Yes | No |  | Yes | No |  |
| --- | --- | --- | --- | --- | --- | --- | --- | --- |
| X |  | On Site at UMMS/UMMMC |  | X | Adults |  | X | Questionnaires (please provide) |
|  | X | Multicenter Study |  | X | Pregnant Women |  | X | Filming/video/audio |
|  | X | Cooperating Institutions | X |  | Minors (under 18) | X |  | Marketed drugs |
| X |  | Research Currently Funded | X |  | Teenagers ( 12-17) |  | X | Diagnostic Radiation |
|  | X | Financial interest involved |  | X | Prisoners |  | X | Therapeutic Radiation |
| X |  | Funding applied for |  | X | Fetuses / Abortuses |  | X | MRI |
|  | X | UMMMC inpatients | X |  | Randomization |  | X | Ultrasound |
| X |  | UMMMC outpatients | X |  | Placebo |  | X | Radioisotopes |
|  | X | Normal volunteers |  | X | Investigational drugs/device |  | X | Radiation involved? |
|  |  | Other |  | X | Increased hospital costs |  | X | Would receive radiation regardless |
| X |  | Males |  | X | Mental Impairment |  | X | Radiation Safety Approval Needed? |
| X |  | Females |  | X | Data bank |  | X | Biosafety Review Needed? |
|  | X | Phase I Study |  | X | Phase II Study | X |  | Phase III Study |

**SECTION III**

PRINCIPAL INVESTIGATOR’S ASSURANCE

As Principal Investigator for this study, I acknowledge and accept my responsibility, as mandated by the UMMS Assurance of Compliance for Protecting the rights and welfare of the human subjects taking part in this research study.

Assuring that the risks to an individual are outweighed by the potential benefits to him/her or by the importance of the knowledge to be gained.

Complying with all the applicable requirements specified by the UMMS Institutional Review Board as a condition of IRB approval.

Completing the required education either by reading the Guidelines for the Protection of Human Subjects in Research and subsequently answering at least 25 out of 28 questions correctly on the true/false, multiple choice, Human Subjects Exam **or** by completing the required modules for the CITI Course in the Protection of Human Research Subjects.

Providing each research subject with a signed copy of the IRB-approved consent form at the time of consent.

Retaining the original signed forms in a reasonably secure and confidential area for at least three years after termination of the research project.

Obtaining approval from the UMMS IRB of any proposed changes in a previously approved study. The proposed changes will not be implemented before IRB review and approval, unless necessary to eliminate apparent immediate hazards to subjects.

Informing the IRB immediately if I become aware of any violations of HHS regulations (45CFR46), FDA regulations (21CFR50, 56) or IRB requirements for the protection of human subjects.

Submitting progress reports of approved research as often as, and in the manner prescribed by, the UMMS IRB (the frequency of these will be on the basis of risk to subjects, but will be at least annually).

Within 48 hours (in-house events) or five working days (sponsor-reported events) report any unanticipated serious adverse experiences, injuries, and other unanticipated problems that involve risks to subjects and others, either physical, psychological, or threats to privacy.

Reporting any research subject’s death within five working days, regardless of cause.

Understanding that the failure to comply with all applicable HHS and FDA regulations, IRB requirements/policies, and the provisions of the protocol as approved by the IRB may result in suspension or termination of my research project.

**The Principal Investigator’s signature must be obtained before submitting.**

Signature of Principal Investigator: Date: 9-7-10

Type PI name and title: Benjamin U. Nwosu, MD

**SECTION IV**

DEPARTMENTAL / DIVISIONAL APPROVAL

**I have reviewed the attached research project for both ethical considerations and technical merit and recommend its approval.**

**I certify that there are adequate resources and facilities to carry out this research, including staff, funding, space, recordkeeping capability, and resources to address serious adverse events and possible research-related injuries.**

Signature of PI’s Department Chair: Date:

Type Name and title of Chair: Marianne E. Felice, MD

Signature of PI’s Division Chief: Date:

Type Name and title of Division Chief: Mary M. Lee, MD

Will this research involve faculty or recruit patients from another department besides the department listed above?

| Yes |  | No | X |
| --- | --- | --- | --- |

**If yes, please complete**

MY SIGNATURE BELOW INDICATES THAT I AM AWARE OF AND AGREE TO INVOLVE MY DEPARTMENT IN THIS RESEARCH PROJECT

Department Name: Pediatrics

Department Chair Name: Marianne E. Felice, MD

Signature of Department Chair: Date:

Please “X” boxes below that apply

| Faculty from my department will be involved in this research study | X |
| --- | --- |
| Patients from my department will be involved in this research study | X |

The section above may be duplicated if there is more than one additional department or faculty member from another department that is participating in this study.

Will this research involve faculty or recruit patients from another department besides the department listed above?

| Yes |  | No | X |
| --- | --- | --- | --- |

**If yes, please complete**

MY SIGNATURE BELOW INDICATES THAT I AM AWARE OF AND AGREE TO INVOLVE MY DEPARTMENT IN THIS RESEARCH PROJECT

Department Name:

Department Chair Name:

Signature of Department Chair: Date:

Please “X” boxes below that apply

| Faculty from my department will be involved in this research study |  |
| --- | --- |
| Patients from my department will be involved in this research study |  |

The section above may be duplicated if there is more than one additional department or faculty member from another department that is participating in this study.

**SECTION V**

**DESCRIPTION OF RESEARCH PROJECT**

**1**. **PERSONNEL ENGAGED IN THE RESEARCH STUDY.** List all personnel engaged in the study. This list must agree with that in Section VI (Delegation of roles/responsibilities).

1. Benjamin U. Nwosu, MD

2. Louise Maranda, PhD

3. Karen Cullen, RN, CDE

4. Lisa Greenman, RD

5. Amanda Angelescu, MD

6. Olga T. Hardy, MD

7. Michael Stalvey, MD

8. Penny Kadmon, MD

9. Leslie Soyka, MD

10. Mary M. Lee, MD

**2. GENERAL STATEMENT OF PROBLEM**

Purpose: Include concise hypothesis to be tested by proposed research.

The significance of this project is to investigate the effects of adjunctive metformin therapy in children and adolescents with double diabetes.

There is a paucity of data on the role of adjunctive metformin therapy in children and adolescents with double diabetes. To help fill this knowledge gap, we propose a randomized, double-blind, placebo-controlled trial to evaluate changes in hemoglobin A1c (HbA1c) and anthropometry in patients with a diagnosis of type 1 diabetes who also have features of type 2 diabetes or metabolic syndrome as well as patients with type 2 diabetes who possess diabetes-associated autoantibodies. This will help determine the safety profile, and efficacy of adjunctive metformin therapy in these subjects.

**Hypothesis:**

The addition of an insulin-sensitizing agent such as metformin to a protocol-driven, patient-directed, treat-to-target insulin regimen will improve glycemic control more rapidly in youth with poorly-controlled double diabetes.

**Aims:**

**Primary Aim**: We will investigate the efficacy of hemoglobin A1c reduction by a protocol-driven, patient-directed, treat-to-target insulin regimen plus metformin vs. protocol-driven, patient-directed, treat-to-target insulin regimen plus placebo in youth with poorly-controlled double diabetes.

**Secondary Aims:**

(1) We will measure the changes in A1c levels as patients transition from standard insulin therapy (run-in period) using aspart and detemir to treat-to-target insulin therapy using the same insulins (aspart and detemir).

(2) Given the insulin-sensitizing effect of metformin, we will also investigate the magnitude of HbA1c normalization in patients on a combination of treat-to-target insulin regimen plus metformin therapy.

(3) We will compare the effects of treat-to-target insulin regimen plus placebo vs. treat-to-target insulin regimen plus metformin on the following outcome measures: blood pressure, waist circumference, body mass index, lipid profile, adiponectin, leptin, and 25-hydroxyvitamin D in children with double diabetes. Leptin and adiponectin are adipocytokines which serve as surrogate biomarkers of adiposity and possible cardiovascular endpoints.

Background and RationalE:

The significance of this project is to determine the efficacy of hemoglobin A1c reduction by patient-directed, treat-to-target insulin regimen and adjunctive metformin therapy in children and adolescents with **double diabetes**.

A. Introduction and Scope of the Problem

**Childhood obesity** is now epidemic in the United States. According to the most recent NHANES data, about 16% of children and adolescents have a body mass index (BMI) (kg/m2) ≥95^th^ percentile for age and gender [1] [Fig 1]. Body mass index of >95^th^ percentile is classified as overweight by the Center for Disease Control and Prevention [2,3], and as obesity by European criteria [4].

The prevalence of obesity has tripled in the past three decades [5] among male and female adolescents, and across different racial and ethnic groups[5-7]. There has also been a concomitant increase in the prevalence of many obesity-related co-morbid conditions[8] such as type 2 diabetes (T2DM), dyslipidemia, hypertension, obstructive sleep apnea, poor quality of life and mortality in adulthood [9-12]. Although obesity is associated primarily with T2DM due to insulin resistance, [13], it may also impact type 1 diabetes morbidity.


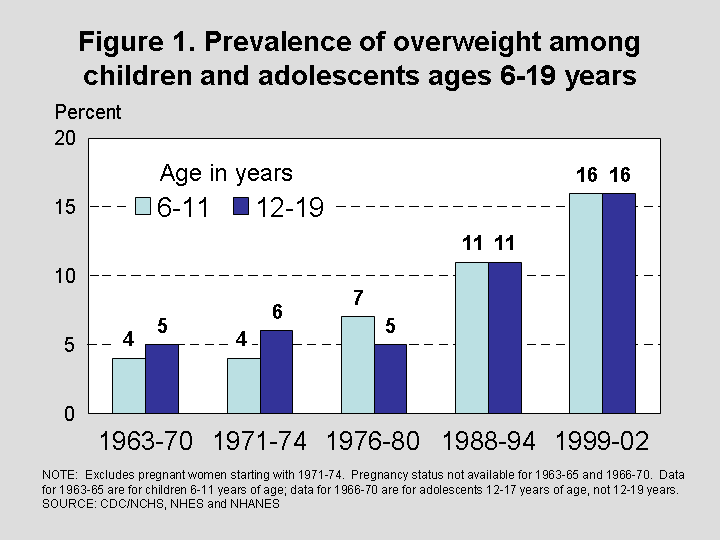


Figure 1. Prevalence of obesity among children and adolescents ages 6-19 years. Source CDC/NCHS, NHES and NHANES[1].

Type 1 diabetes (T1DM) is caused by autoimmune destruction of the beta cells of the pancreas leading to insulinopenia. It is sub-classified into 2 main categories- type 1A and 1B[14]. In type 1A, individuals have one or more of the anti-islet cell (including glutamic acid decarboxylase, and insulinoma antigen-2) or anti-insulin antibodies. In type 1B these antibodies are absent, but the clinical and biochemical features are similar to 1A. Type 2 diabetes is characterized by insulin resistance and absence of diabetes-associated antibodies in serum.

A new subset of diabetes, called **double diabetes** is becoming increasingly prevalent as a result of the epidemic of childhood obesity[15-17]. In double diabetes, elements of both type 1 and type 2 diabetes co-exist in the same person. In this condition, **individuals with type 1 diabetes have the insensitivity to insulin that is most often associated with obesity; and individuals with type 2 diabetes have antibodies against the pancreatic beta cells**[13] (Figure 1). Unlike type 1 and type 2 diabetes, there is no consensus on the therapeutic modalities for double diabetes.

### Double Diabetes: Definition and Summary[18]

When a person has elements of both type 1 diabetes and type 2 diabetes, they have "double diabetes" (or hybrid diabetes). This occurs when:

- A child with type 1 diabetes becomes overweight and develops the basic feature of type 2 diabetes and obesity – the body becomes resistant to insulin.
- A child with type 2 diabetes has one of the key features of type 1 – the presence of antibodies in the blood against the insulin producing beta cells of the pancreas causing a decrease in the body's ability to produce insulin.

### What Does Double Diabetes Look Like In Someone With Type 1 Diabetes?

In someone with type 1, the signs and symptoms typical of type 2 diabetes can develop gradually. How quickly they develop depends mostly on a person's genes and their degree of weight gain.

- They are usually overweight or obese.
- They need a high dose of insulin to control their blood sugar.
- They have developed [insulin resistance](http://www.childrenwithdiabetes.com/dictionary/i.htm#Insulin%20Resistance), a condition where the body's cells no longer respond normally to insulin that the pancreas produces or that is given by injection.
- They may have high blood pressure or high cholesterol.
- They may have an abnormal lipid profile and poor diabetes control.
- Teenage girls and women may have the [polycystic ovary syndrome](http://www.childrenwithdiabetes.com/dictionary/p.htm#PCOS) which includes several hormonal abnormalities that lead to infrequent or absent periods, and excess hair growth and acne.

### What Does Double Diabetes Look Like In Someone With Type 2 Diabetes?

If a teenager or child with all of the typical clinical features of type 2 diabetes –- excess body weight, [acanthosis nigricans](http://www.childrenwithdiabetes.com/dictionary/a.htm#acanthosis) (velvety and dark colored skin of the neck, armpit and groin), high blood pressure, high cholesterol, polycystic ovary syndrome, positive family history of type 2, belonging to ethnic/racial minority group -– has the presence of [antibodies](http://www.childrenwithdiabetes.com/dictionary/a.htm#Antibodies) against the insulin producing beta cells of the pancreas, we would say he or she has elements of both kinds of diabetes. A number of reports have described that as many as 15-20% of teens with the typical symptoms of type 2 diabetes have antibodies circulating in their blood. Because of the presence of these antibodies, they can no longer be considered a pure type 2 case, as shown below in Figure 2.

**Figure 2. The relationship between T1DM, T2DM and Double Diabetes**


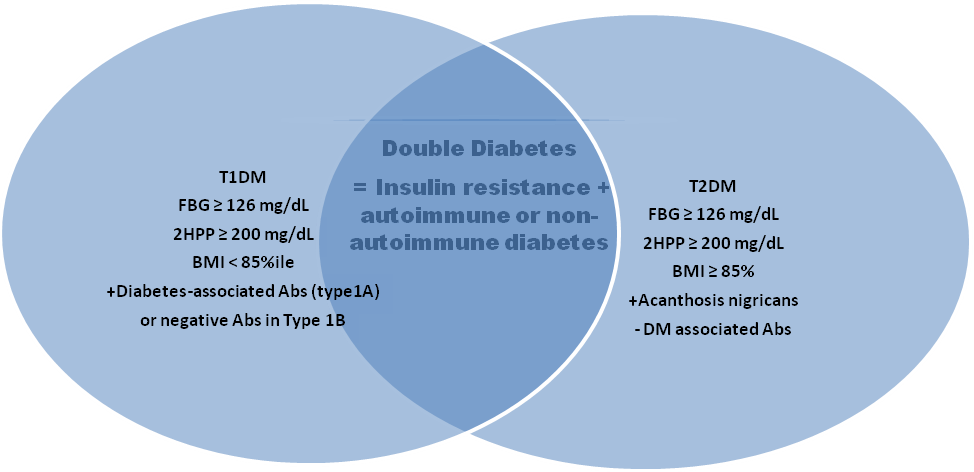


TIDM=type 1 diabetes, T2DM = type 2 diabetes, FBG=fasting blood glucose, 2HPP= 2 hour post prandial glucose level; BMI=body mass index; Abs= antibodies

The incidence of both type 1 and type 2 diabetes is rising in children and adolescents[13]. Data from the EURODIAB study indicate that the overall prevalence of type 1 diabetes among young people under 15 years is increasing by greater than 3% each year, and by more than 6% a year in children aged up to four years [19].Analysis of the 2002 to 2003 data from SEARCH for Diabetes in Youth, a multicenter study funded by the Centers for Disease Control and Prevention (CDC) and the National Institutes of Health (NIH) to examine type 1 and type 2 diabetes among children and adolescents in the United States, showed that 15,000 youth in the United States are newly diagnosed with type 1 diabetes annually, and about 3,700 youth are newly diagnosed with type 2 diabetes annually. The reported rate of new cases among youth was 19 per 100,000 each year for type 1 diabetes, and 5.3 per 100,000 for type 2 diabetes [20].

The high prevalence of diabetes imposes a considerable burden on the United States health care budget. In 2007, the Center for Disease Control and Prevention (CDC) reported that 23.6 million Americans had diabetes, and the total cost of managing diabetes in that year was $174 billion[21].

B. Theoretical basis of the study

**The Burden of Poor Metabolic Control in Youth with Diabetes**

The availability of insulin analogs and diabetes monitoring devices has improved diabetes care in the United States. However, according to recent studies, the prevalence of poorly-controlled diabetes in youth is still high. This poor glycemic control predisposes the youth to acute and chronic complications of diabetes, which will in turn impose a huge burden on the national health care budget.

A recent publication by SEARCH for Diabetes in Youth Study group showed that a high proportion of youth with diabetes had poor HbA1c values, with 17% of the youth with TIDM, and 27% of those with T2DM showing poor control, defined as HbA1c ≥ 9.5%[22]. The American Diabetes Association target values for HbA1c in relation to age are as follows: 7.5-8.5% at age < 6 years, <8% at age 6-12 years, <7.5% at age 13-18 years, and <7.0% at age 19+ years[23]. Thus only a minority of children and adolescents are meeting the recommended glycemic targets.

There are physiological, therapeutic, sociodemographic, genetic, and environmental reasons for poor metabolic control in children and adolescents with type 1 diabetes.

**Genetic and Environmental Factors:**

The epidemic of childhood obesity has led to increased diagnosis of metabolic syndrome and type 2 diabetes in all children including those with existing type 1 diabetes[24]. This confluence of pathophysiologies underlying type 1 and type 2 diabetes is referred to as ‘double diabetes’. Obese or overweight children have been reported to develop type 1 diabetes at younger ages than children of normal weight[24]. The recently published SEARCH for Diabetes in Youth Study[26] reported an obesity prevalence rate of 12.6% in US youth with type 1 diabetes. The study also reported a higher prevalence of overweight (BMI 85th – 95th percentile) among youth with type 1 diabetes than in those without diabetes (22.1% vs. 16.1%) (P<0.05). Some children with type 1 diabetes have either a first- or second-degree relative with type 2 diabetes. Orchard et al[27,28], in the Epidemiology of Diabetes Complications Study, reported that patients with type 1 diabetes who have a positive family history of type 2 diabetes were at greater risk for cardiovascular disease than those who did not. Data from the Diabetes Control and Complications Trial (DCCT) show that weight gain and central obesity are associated with insulin resistance, hypertension, and dyslipidemia in type 1 diabetes[29], and data from Epidemiology of Diabetes Interventions and Complications (EDIC) Study show that central obesity is an independent risk factor for incident microalbuminuria in individuals with type 1 diabetes[30]. However, both DCCT and EDIC follow up studies show that intensive diabetes therapy results in a uniform, major reduction in (and significant protection from) microvascular disease[31], even in overweight or obese type 1 diabetes patients[28].

Obese/overweight type 1 diabetic children require a large amount of insulin to achieve good control. Such children are at risk for insulin resistance, and their existing type 1 diabetes predisposes them to dyslipidemia, hypertension, and other components of the metabolic syndrome[32]

**Physiological Factors:**

The physiological factors that contribute to poor glycemic control in youth are in part related to the hormonal changes in puberty. Puberty is associated with relative insulin resistance, reflected in a two- to threefold increase in the peak insulin response to oral or intravenous glucose[33]; insulin-mediated glucose disposal is approximately 30% lower in adolescents than in prepubertal children or young adults[34]. This physiologic insulin resistance of puberty is of minimal consequence in the presence of adequate beta-cell function[35]. The cause of this physiologic resistance is likely the transitory increased activity of the growth hormone-insulin growth factor axis, as well as sex steroids, which coincides with the physiologic insulin resistance of adolescence[36] and act as counter-regulatory hormones.

Furthermore, weight gain is prevalent in adolescents with type 1 diabetes after attainment of final height, which might further impair insulin sensitivity[37]. Excessive weight gain causes insulin resistance by various mechanisms[38]. Visceral fat liberates large amounts of non-esterified fatty acids, which stimulate neoglucogenesis in the liver and diminish glucose uptake in the muscles. Obesity is associated with increased activity of the sympathetic nervous system, which in combination with direct release of tumor necrosis factor, resistin and other adipocytokines contribute to insulin resistance. Local intramyocellular triglyceride accumulation may also play a role. Insulin dosages are often increased to overcome the resistance to insulin, but metabolic control still frequently worsens during the later stages of pubertal development[37].

**The Sociodemographic and Therapeutic Factors:**

The sociodemographic and therapeutic factors associated with poor glycemic control in youth were outlined in the recently published SEARCH for Diabetes Study in Youth. These factors include race/ethnicity, socioeconomic status, parental education, parental involvement in diabetes management, family dynamics, and whether a patient receives care from an endocrinologist[22]. Chalew et al[39] reported higher mean HbA1c levels in African American children independent of sex, insurance status, BMI, and number of clinic visits. In contrast, however, Gallegos-Macias et al[40] reported that the higher HbA1c values seen with Hispanic youth with TIDM compared with non-Hispanic youth with TIDM were accounted for by lower socioeconomic status irrespective of race/ethnicity. Petitti et al[22] reported that lower parental education level and living in a single-parent home were associated independently with worse glycemic control.

**Clinical Trials and Therapeutic Interventions:**

The increasing insulin resistance and deterioration of glycemic control in adolescents create a great need for alternative therapeutic strategies in adolescents with type 1 diabetes. One such strategy is the addition of a drug that improves insulin sensitivity. Metformin is a biguanide, which acts principally by increasing insulin sensitivity in the liver by inhibiting hepatic gluconeogenesis and thereby reducing hepatic glucose production[41]. Other minor mechanisms include decreasing fatty acid oxidation and intestinal glucose absorption[42], and increasing peripheral insulin sensitivity by enhancing glucose uptake in the muscles[43]. Metformin has mainly been used in adult patients with type 2 diabetes and several studies have shown beneficial effects on body weight, blood lipid levels and metabolic control[44-46]. Randomized controlled trials with metformin in adolescents with type 2 diabetes reported an improvement in fasting plasma glucose[47]. However, there have been conflicting reports from studies in adolescents with type 1 diabetes [41-43,48,49]. The benefit was transient in one study[49] and negative in another[48]. The main drawback of these studies was the small sample size and lack of reporting on long term benefit and safety of adjunctive therapy in many of them[50].

There are only 2 randomized, placebo-controlled trials of adjunctive metformin therapy in adolescents with type 1 diabetes. In a randomized placebo controlled trial in children with type 1 diabetes who were treated for 3 months with adjunctive metformin, Sarnblad et al[43] reported a significant decrease in A1c from 9.6% to 8.7%(p<0.05) in the metformin group, compared to 9.5 to 9.2% (p=NS) in the placebo group. In another study, Hamilton et al[41] reported an HbA1c 0.6% lower in the metformin group than in the placebo group (P<0.035), after 3 months of therapy. Mean HbA1c at the end of the study was decreased by 0.3% in the metformin group, while it increased by 0.3% in the placebo group (p=0.03). Both studies reported no difference in mean body mass index and serum lipids in the metformin versus placebo group after 3 months of therapy. Hamilton et al[41] reported no significant changes in mean insulin sensitivity, measured by frequently sampled glucose after intravenous glucose tolerance test, after 3 months of metformin therapy in the metformin versus placebo group. Sarnblad et al[43], using hyperinsulinemic euglycemic clamp study, demonstrated no significant change in insulin sensitivity after 3 months between the groups, but they did report an increase in insulin sensitivity in the metformin group during the study (P<0.05). Hamilton et al[41] reported a significant change in the mean daily insulin dose in the metformin group in comparison to the placebo group after 3 months of metformin therapy of -0.14 vs. 0.02, P=0.01. However, Sarnblad[43] did not find a significant difference in the daily insulin dosage between the metformin and placebo groups after 3 months of therapy (1.1 vs. 1.3).

C. Gaps in current knowledge

**Double Diabetes**

The two randomized, controlled studies by Hamilton and Sarnblad did not categorically recruit children and adolescents with double diabetes. This is important because this sub-set of diabetic youth is known to be insulin resistant and may require a careful titration of insulin doses. Adjunctive metformin therapy to achieve glycemic control may also be more effective in this subset of diabetes patients. Our aim is to primarily study this group of patients to determine the role of protocol-driven, treat-to-target regimen alone or in combination with metformin therapy in their care.

Metformin is approved by the Food and Drug Administration for use in children with type 2 diabetes, and recently it has been recommended that metformin added to insulin therapy might be used in clinical practice in adolescents with type 1 diabetes who are poorly controlled and show evidence of insulin resistance (double diabetes) as seen in type 2 diabetes[50].

**Treat to Target Regimen**

Even though the randomized controlled studies by Hamilton and Sarnblad were designed to compare the effectiveness of adjunctive metformin therapy compared to insulin therapy alone, they were not designed to compare metformin adjunctive therapy to protocol-driven, optimized insulin therapy. Neither study demonstrated a strong head-to-head comparison of adjunctive metformin to patient-directed, treat to target insulin regimen to ensure optimal insulin delivery during the study. Such a comparison is critical because poor glycemic control contributes to insulin resistance[51] as there is an inverse relationship between glycemic control (as determined by HbA1c) and insulin sensitivity (estimated by glucose infusion rate during euglycemic-hyperinsulinemic clamp)[52].

Evidence suggests that many patients often do not have insulin doses titrated sufficiently to achieve target levels of glucose control[53,54]. These patients remain on suboptimal doses of insulin and fail to reach treatment targets[55]**.** In a recent study Blonde et al[55] demonstrated the efficacy of algorithm-guided, patient titration of once daily long acting insulin in normalizing HbA1c in adult patients with type 2 diabetes. They conducted a 20-week, randomized, controlled, open label, multicenter, parallel-group study comparing the safety and efficacy of insulin detemir administered once daily in combination with oral antidiabetic agents when titrated to two fasting plasma glucose targets ( 3.0-5.0 mmol/L versus 4.4.-6.1 mmol/L) for the treatment of type 2 diabetes in adults. In that study, fasting plasma glucose decreased throughout the first 8 weeks of the study and then generally remained flat for each treatment group. The combined treatment groups achieved a mean HbA1c level of 6.9% at the end of the study. There were significant reductions in HbA1c in both titration groups: in the 3.9-5.0 mmol/L fasting plasma glucose target group, HbA1c values decreased from a baseline mean of 8% to 6.8% at 20 weeks. In the 4.4-6.1 mmol/L fasting plasma glucose target group, HbA1c values decreased from a 7.9% at baseline to 7.0% at 20 weeks. Overall rates of hypoglycemia episodes were low and were comparable between treatment groups: 7.73 and 5.27 events/subject/year for the 3.9-5 mmol/L and 4.4-6.1 mmol/L groups, respectively. Mean weight changes from baseline to the end of the study were small and did not differ significantly between groups.

In this study we will explore the role of **protocol-driven treat-to-target regimen in a special subset of children and adolescents with diabetes: the group with double diabetes.** Given the rising incidence of obesity in the general population we speculate that many children with type 1 diabetes will eventually develop double diabetes. Thus, it is timely to devise an appropriate management protocol to treat this burgeoning sub-population.

This study will be conducted at the UMass Memorial Medical Center, which is located in Worcester County in the state of Massachusetts. It serves the central Massachusetts area and beyond. It caters to the healthcare needs of a community with the following ethnic diversity: 40.7% Caucasian, 12.9% African-American, 7.8 % Asian, 35.7% Hispanic/Latino, 0.5% Pacific Islander and 2.4% Multi-Racial.

At the Children’s Medical Center of the Umass Memorial Medical Center, six pediatric endocrinologists follow 322 patients with type 1 diabetes. We reviewed BMI data on 275 of these patients. Our data showed that of these 275 patients, 61 patients (22.2%) are overweight, and 21 (7.64%) are obese. Thus, there are 82 overweight or obese type 1 diabetes patients in our facility. We see about 2-3 new patients with type 1 diabetes per week. Therefore, we will fulfill our recruitment goal within the allotted time period. National surveys have shown that about 15-20% of patients with type 2 diabetes possess diabetes-associated autoantibodies.

D. Long term benefits and contribution to the scientific field

Blonde et al[55] demonstrated that self-titration regimens facilitate empowerment of patients, allowing them to become more involved in their treatment, which can result in improved glycemic control. Patient-directed insulin titration is increasingly important as health care practitioners often do not have the resources to advise patients with the frequency needed to effectively titrate their insulin doses to maintain euglycemia. Thus, it is clear that patient empowerment is critical for motivation to reach treatment targets.

The role of patient-directed, treat-to-target titration regimen in diabetes management has not been adequately studied in adolescents with type 1 diabetes. Because there are several causes of poor glycemic control in adolescents, a mechanism that engages the participation of the youth and their parents in reaching simple and clearly defined glycemic goals will lead to significant improvements in glycemic management and the prevention of acute and chronic complications of diabetes mellitus. Another major advantage of treat-to-target protocol is the ease of maintaining compliance in the long term with insulin alone, than with a combination of insulin and oral antidiabetic agent in adolescents with type 1 diabetes. Moreover, the long term side effects of metformin therapy in children and adolescents with type 1 diabetes is unknown[56-58]. To clarify this potential advantage of treat-to-target regimen, we will compare the hemoglobin A1c changes during standard therapy to similar changes during treat-to-target regimen.

This research project will contribute to the scientific field in several ways. Given the conflicting reports on the efficacy of adjunctive metformin therapy in adolescents with type 1 diabetes, this double blind, randomized, placebo controlled trial will **demonstrate the effect of meformin on HbA1c reduction under optimized insulin titration regimen.** Secondly, given that most of the available studies were not carried out under optimized insulin treatment regimen, it is unclear whether a titrated insulin regimen would have a superior-, or similar effect to combined metformin and insulin regimen in type 1 diabetes patients, and whether it will be beneficial compared to standard insulin therapy. Moreover, because the long term side effects of biguanides and efficacy as an adjunctive agent in children with type 1 diabetes are not known, it is necessary to study **novel approaches to insulin management** **(such as patient-directed, treat-to-target regimen)** in this population. Finally, children and adolescents who are allergic to metformin, or have hepatic and renal diseases, will be unable to receive adjunctive metformin therapy. Thus, this category of children and adolescents with double diabetes will require a protocol-driven, treat-to-target regimen to maintain euglycemia.

b. Please describe the Investigator’s previous work on the problem.

Dr Nwosu is a sub-investigator on the NIH-funded Treatment Options for type 2 Diabetes in Adolescents and Youth (TODAY) study. This is a multi-center clinical trial with the aim of determining the best therapeutic strategies for type 2 diabetes in youth.

c. What are the aspects that justify the use of human subjects, human data, or specimens as part of this research?

This study is designed to evaluate the effects of adjunctive metformin therapy on A1c and body mass index in children and adolescents with double diabetes. Thus, we can only achieve our goal by investigating these effects in human subjects.

d. Attach references as appropriate.

(See below).

**4. DETAILED DESCRIPTION OF RESEARCH PLAN (especially as it affects the subject)**

1. Include a schematic representation of what the research will entail (e.g. a table with the number of visits and what will happen at each visit or flow diagram of subject’s involvement over time).

Table 1. Schematic Representation of the Study Protocol for Trial of optimized Insulin-Metformin Therapy

| Procedure | Visit I  (-3mo): Start of Run-in period +  screening  Duration: 1hr | Visit II (-2 mo)  Run-in period+ | Visit III (month 0)  Baseline study visit: Duration 1hr | Visit IV (month +3)  Interim study visit:  Duration 30 min | Visit V (month +6)  Interim study visit;  Duration 30 min | Visit VI  (month +9)  Study conclusion  Duration:1hr |
| --- | --- | --- | --- | --- | --- | --- |
| History and Physical Exam | X | X | X | X | X | X |
| Nutrition Counseling |  | X | X | X | X |  |
| Instructions on treat-to-target regimen | X | X | X | X | X |  |
| Research Blood draw |  |  | X | X | X | X |
| Urine collection | X |  |  |  |  | X |
| Screening labs  (part of research lab draw) | X |  |  |  |  |  |
| Run in phase | X | X |  |  |  |  |
| Implementation of Treat-to-target regimen | X | X | X | X | X |  |
| Metformin/Placebo |  |  | X | X | X |  |
| Review of blood glucose data and insulin dose adjustments | X | X | X | X | X | X |

Trial design:

**Type of Trial**

1. STUDY DESIGN AND SETTING

We propose to use a randomized, placebo-controlled, double blind study design to determine the efficacy HbA1c reduction by treat to target regimen plus metformin versus treat to target regimen plus placebo in pediatric patients of ages 10-18 years with poorly controlled double diabetes.

A 3-month run-in period will precede the interventional phase of the study. All patients will be placed on standard aspart and detemir regimen during the run-in phase. Both insulin aspart and insulin detemir are commonly prescribed to treat type 1 and type 2 diabetes. **Insulin aspart** (marketed by [Novo Nordisk](http://en.wikipedia.org/wiki/Novo_Nordisk) as "NovoLog/NovoRapid") is a fast acting [insulin analogue](http://en.wikipedia.org/wiki/Insulin_analogue) with a rapid onset of action within 15 minutes and a duration of action of about 2-3 hours. It is used to cover meal time insulin requirements. **Insulin detemir** is a long-acting human [insulin analogue](http://en.wikipedia.org/wiki/Insulin_analogue) for maintaining the basal level of insulin. [Novo Nordisk](http://en.wikipedia.org/wiki/Novo_Nordisk) markets it under the trade name **Levemir**. Its onset of action is approximately two hours and its duration of action is 24 hours. Unlike NPH insulin, it has no peak, thus delivers a steady amount of insulin throughout the day.

**Run-in Phase**: This is the period of preparation before the actual study starts. During the run-in period all participants will be transitioned from their existing insulins to detemir and aspart, without instituting treat-to-target regimen, or adding metformin or placebo during the run-in phase. The transition from non-determir long-acting insulin to detemir insulin will be based on 1:1 insulin conversion.  Specifically, we will strive to transition patients to the same dose of detemir as they were on their prior long acting insulin; and also maintain a basal : bolus insulin ratio of 1:1, i.e., each component (basal and bolus insulin dose) will constitute 50% of the total daily dose of insulin. The insulin boluses for meals will be calculated from an insulin to carbohydrate ratio that would be predetermined by the endocrinology team for each patient.

This will be followed by close monitoring of blood glucose levels in the first 2 weeks on standard aspart and detemir regimen to ensure normoglycemia. Parents will be advised to fax a copy of their child’s **glucose log book** in the first 2 weeks for our review and recommendation of insulin dose adjustment if necessary. All subjects will continue to use their personal log books and logging techniques. All participants will return to the clinic at the end of one month (from the time of starting on standard aspart and detemir regimen) for a comprehensive review of glycemic management.

At the end of the 3-month run-in period, all participants will continue on treat-to-target insulin regimen, and will then be randomized to either of the 2 arms of the study: **an experimental arm**, consisting of treat-to-target insulin regimen plus metformin, and **a control arm** consisting of treat-to-target insulin regimen plus placebo. Both the physicians and patients will be blinded to the oral agents being administered to patients.

**Summary of Procedure:**

This research study involves 6 visits: two screening visits and 4 study visits. Subjects who meet the inclusion criteria will be invited to enroll in the study. **Consent** will be obtained from subject’s parents or guardian. Participants who are 10-17 years of age will give assent; and participants who are 16 and 17 years of age will sign the parental consent form along with their parents. Subjects will undergo a 3-month **run-in period** to screen for complications, to update patient education, and to assess subject’s compliance with study protocol. To **screen for complications** of therapy, we will review patients’ history of frequency and severity of hypoglycemic events. Patients with a history of severe hypoglycemia (<30 mg/dL) on more than 3 occasions in the preceding month will have to be closely monitored. Specifically, we will review each episode of severe hypoglycemia immediately after the event to determine the cause. Common causes include overinsulinization, administering the wrong type of insulin, skipping meals, and unexpected strenuous exercise. If our review detects any of these common causes, we will instruct the family on how to prevent future occurrences and monitor the patient’s glucose logs every 3 days for any repeat hypoglycemic events. If our review did not detect any of these causes, then we will recommend that the candidate be withdrawn from the study.

Patients with hypoglycemia unawareness will only be enrolled after they have regained the ability to recognize hypoglycemic events. This is easily achieved by allowing these individuals to main slightly elevated blood glucose levels for a period of 2 weeks. Patients will be placed on detemir and aspart with no adjunctive metformin or placebo during the run-in period. All participants will be instructed on the treat-to-target regimen and be given a **summary sheet** to familiarize themselves with the regimen. Treat-to-target regimen will only commence at the end of the run-in phase.

*Consent:*

After the investigator explains the study and answers all questions, 10-15 year old participants who agree to participate will sign an assent form, 16-18 year old participants who agree to participate will sign an informed consent form, and a parent of the 10-17 year old participants will provide permission by signing the informed consent form.

*History:* To ensure eligibility for the study, we will take a detailed history of subject’s medical condition. This history will focus on patient’s current medical diagnosis, past medical and birth history, social history. We will document any family history of type 1 or type 2 diabetes, obesity, and related comorbidities. We will also document subject’s medications, supplements, diet and exercise plan.

*Physical Examination:* All participants will undergo a detailed physical examination at -3mo, -2mo, baseline, and at 3 month intervals throughout the duration of the study.

*Screening Laboratory Test:* All participants will undergo a screening study consisting of liver function tests, serum creatinine, thyroid function tests, and celiac antibody panel.

*Urine Sample:* A spot urine sample will be collected for microalbumin and creatinine at baseline and at study conclusion (visit #6). Urine pregnancy test will be obtained on all female participants and will be repeated at baseline, +3mo, and + 6mo.

*Run-in phase:* All participants will be transitioned to detemir and aspart, without instituting treat-to-target regimen, or adding metformin or placebo.

At visit 2, all participants will return to the clinic one month after the start of the run-in phase for a brief physical examination, review of glucose data, and insulin dose adjustments. We will also review the protocol for patient-directed, treat-to-target regimen with the families.

*Nutrition Counseling*: All subjects will undergo nutrition counseling by a board certified dietician at -2, 0, +3, and at +6 months.

At Visit 3, the baseline intervention will begin at month ‘0’. Following the screening study, qualified subjects will return to the Children’s Medical Center of the Umass Memorial Medical Center in the morning, after an overnight fast for initial intervention study blood draw. Anthropometric measurements: height, weight, waist circumference will be obtained. Vital signs and fasting glucose level will be checked. A venous catheter will be inserted in the antecubital fossa and blood sample will be drawn for fasting lipids, leptin, adiponectin, hemoglobin A1c, serum creatinine, hepatic function tests, and 25-hydroxyvitamin D at baseline. These analytes will be redrawn at 3, 6 and 9 months.

Following the blood draw, each subject will be started on a patient-directed, treat-to-target insulin regimen. All participants will be on multiple daily injections (MDI) consisting of once daily injection of long acting insulin analog, detemir, meal time insulin boluses with aspart, and a correction factor for insulin to ensure euglycemia between meals. All insulins will be administered subcutaneously using a flexpen device.

Randomization for this study will be done by the Investigational Drug Services (IDS) using www.randomization .com. Randomization will be 1:1 (metformin : placebo) and will be blocked for every 10 subjects. Pharmacy will maintain blinding information and PI will contact IDS for emergency unblinding. IDS is available 24 hours a day and 7 days of the week via pager. Call schedule is available on OurNet.

Subjects will be followed with twice weekly phone calls in the first month of enrollment to assess compliance and to help calculate insulin titration doses and make necessary dose adjustments. After the first month of study, subjects will be contacted every 2 weeks until the end of the study.

Each subject will be provided with a **glucometer** and will be instructed on how to download the glucose readings to a computer every third day and email or fax the downloaded data to a contact number or email address provided by the investigators for review and a follow up recommendation. Investigators will review glucometer readings on the same day that they are emailed or faxed. All participants will return to the clinic at +3 and +6 months for interim visits and at +9 months for study conclusion.

b. Inclusion/Exclusion Criteria - As appropriate, explain what steps will be taken to insure that subjects meet the criteria (e.g. healthy, not pregnant, etc).

Subjects who meet the following criteria will be enrolled in the study:

**Inclusion criteria:**

1. **General inclusion criteria**
2. Ten to 18 years of age.
3. Pubertal (Tanner stages 2-5, by examination).
4. Hemoglobin A1c level of > 8.0% in the 6 months prior to enrollment.
5. All subjects must have access to a computer.
6. **Specific inclusion criteria: [Subjects could have either #1, or #2].**
7. Subjects with clinical and biochemical features of T2DM of > 6mo duration who also have positive T1DM antibodies
   1. Clinical features: acanthosis nigricans, BMI >85%
   2. Biochemical: evidence of insulin resistance at diagnosis
      1. fasting insulin >27 uIU/mL(normal range 6-27) at a fasting blood glucose of ≥ 126 mg/dL, or
      2. fasting c-peptide level of > 7.1 ng/mL (normal range 0.9 – 7.1), or
      3. Homeostasis model of insulin resistance of >3.16
8. Patients with T1DM of > one yr duration with BMI >85%
   1. Presentation with ketoacidosis at diagnosis
   2. C-peptide <0.9 ng/mL (normal range 0.9 – 7.1),or (insulin < 6 uIU/mL) (NR 6-27) at diagnosis (when blood glucose is ≥ 126 mg/dL)
   3. Can be antibody positive or negative
   4. Increased insulin requirement (>2 Units/kg/day)

**Exclusion Criteria**

1. Subjects on weight altering medications, such as orlistat.
2. Subjects with eating disorder
3. Subjects on medications other than insulin and or metformin that may affect blood glucose level.
4. Subjects with abnormal hepatic function tests.
5. Subjects with nephropathy, defined in this case as an overnight albumin excretion rate of >200 mcg/min using a first morning urine sample collection.
6. Subjects with recurrent diabetes ketoacidosis (more than 2 episodes in the past 12 months), or recurrent severe hypoglycemia (more than 2 episodes of hypoglycemia with altered level of consciousness, requiring assistance to treat in the past year).
7. Pregnant, breast-feeding or the intention of becoming pregnant or not using adequate contraceptive measures.
8. Known or suspected allergy to metformin.
9. The receipt of any investigational drug within 6 months prior to this trial.
10. Active malignant neoplasms.
11. No access to a computer.
12. Subjects currently taking metformin for clinical purposes are not eligible to be enrolled in this study.

***Withdrawal Criteria***

A subject must be withdrawn from the study if any of the following applies:

1. Pregnancy or intention of becoming pregnant
2. Allergic reaction to metformin
3. Subject decides to withdraw from the study*.*
4. Subject may also be withdrawn from the trial at the discretion of the Investigator if judged non-compliant with trial procedures or due to a safety concern.
5. increasing hypoglycemia

***Rationale for Trial Population***

Because of the rising incidence of childhood obesity, and the increasing prevalence of double diabetes in the population, participants aged between 10 and 18 years will provide the best age range to study the effects of treat-to-target regimen plus placebo or metformin on HbA1c and fasting plasma glucose in children, adolescents and young adults.

c. Discuss the number of experimental and control subjects, and explain the statistical basis for the numbers.

**Subjects:** Two hundred subjects will be screened for this study. Of these, 74 subjects will be randomized to either the placebo or metformin arm of the study.

**Sample size calculation and Statistical Analysis:**

All patients will undergo a 3-month run in phase, during which they will be on detemir and aspart only, with no added metformin or placebo. (Please see a detailed explanation of the run-in phase under Trial Design above). At the end of the run-in period, patients will be assigned to one of the two treatment groups (placebo or metformin) using a random number generation process. Placebo and metformin will be offered in similar form, to ensure participants are blinded to their assignment. Group assignment will be established by a research pharmacist with no direct participation in the study; and principal investigator will not be informed of what group each individual is assigned to.

The primary outcome chosen to measure the impact of metformin on the management of “double diabetes” children is their level of glycosylated hemoglobin (Hb A1c, measured as a %). The secondary outcomes are: a) blood pressure (mmHg), b) waist circumference (cm), c) body mass index (BMI, kg/m^2^), d) lipid profile (LDL and HDL cholesterol and triglycerides (mg/dL)), e) ), leptin (ng/dL), and f) 25-hydroxyvitamin D (ng/mL), g) adiponectin (µg/mL ), and total daily dose of insulin.

All outcomes will be measured on an ongoing basis, at the beginning of the trial, and then at 3 months intervals during the entire study period. The intervention arm of the study will last 9 months. **Sample size calculations** are based on the simple comparison of paired means. Using a confidence level of 95% (Z _(1-α/2)_ = 1.96 for a two-tailed test) and a statistical power of 80% (Z _(1-β)_ = 0.84), calculated group sizes are 29 individuals for each arm of the trial. This number is based on the assumptions that 1) supplementing the treat-to-target protocol with metformin will decrease the Hb A1c level by at least 1.5 percent units, and 2) the standard deviation for Hb A1c levels in obese adolescents is estimated to be 2. Because of the duration of the study, and the possibility of loss to follow-up, a 25% attrition allowance has been made on the ‘n’ of 58, bringing the total number of participants to 74. This is a conservative approach to the sample size calculation, because the repeated measurements will, by construction, improve the power of the study, improving its ability to capture outcomes with more variability than expected.

**Subject Replacement:** To ensure adequate sample size to reach significance according to our power calculations, we will oversample by 25% for each arm of the study. This will enable adequate numbers despite predicted study attrition.

**Statistical analyses** will be conducted so as to capture both the absolute changes in serum values for the proposed outcomes, as well as “time-to-event” information allowing us to compare the 2 groups with maximum efficiency.

1) **H_1A_**: Metformin supplementation of treat-to-target insulin regimen of diabetes care will have an influence on levels of HbA1c over time.

Mean levels of HbA1c will be computed at each time point, applying arcsine transformation to approximate Normality. 95% Confidence Intervals (95% CI) will be constructed around these estimates. All results will be graphed for visual appreciation of possible trends. Consecutive HbA1c levels for patients with and without metformin will be compared using repeated-measures Analysis of variance (ANOVA). Analyses of Covariance will be modeled to account for possible confounding due to gender and age. Similar analyses will be applied to the FPG levels, using the logarithmic transformation instead.

2) **H_1B_**: Patients randomized to adjunctive metformin therapy will achieve a greater reduction in HbA1c and fasting plasma glucose than those on placebo.

Adjunctive metformin and placebo groups will also be compared using both a logistic model and an actuarial survival model for “time-to-event”, where the event of interest is the decrease of the HbA1c level to 8% or less, as the biological cutoff below which serum glucose levels are considered stabilized. Similar analyses will be applied to FPG levels.

3) **H_1C_**: Metformin supplementation of treat-to-target regimen of diabetes care will have a measurable influence on blood pressure, waist circumference, body mass index, lipid profile, leptin, adiponectin, and 25-hydroxyvitamin D.

Mean values for each one of these outcomes will be computed at each time point, applying appropriate transformations where needed to approximate Normality. 95% CIs will be constructed around these estimates. All results will be graphed for visual appreciation of possible trends. Consecutive measurements for patients with and without metformin will be compared using repeated-measures Analysis of variance (ANOVA). Analyses of Covariance will be modeled to account for possible confounding due to gender and age. Non-parametric equivalent of statistical tests will be used where necessary.

4) **H_1D_**: Transition from standard insulin treatment to treat-to-target regimen will provide better diabetes care for all patients. (Please see Table 2 below for a detailed explanation of the protocol for patient-directed forced-titration regimen).

Mean A1c levels at the beginning (-3mo) and at the end (0 mo) of the run-in period in all patients will be compared to subsequent mean A1c levels in the placebo group, using repeated measures ANOVA. Necessary adjustments for possible confounders will be carried out as described above.

d. Does the study involve randomization?

| YesX |  | No |  |
| --- | --- | --- | --- |

**If yes**, please describe process.

**Randomization:**

Randomization for this study will be done by the Investigational Drug Services (IDS) using www.randomization .com. Randomization will be 1:1 (metformin : placebo) and will be blocked for every 10 subjects. Pharmacy will maintain blinding information and PI will contact IDS for emergency unblinding. IDS is available 24 hours a day and 7 days of the week via pager. Call schedule is available on OurNet.

e. How long will each subject be enrolled in the study?

Twelve months.

f. Provide a *brief* overview of what participation in the study will mean to each participant in terms of what he/she will experience. Describe in order, each procedure, how long each procedure will take and how often each procedure will be performed. Include doses & route of administration of any drugs and whether the procedure or drugs would **always**, **sometimes** or **never** be required as part of the subject’s standard of care.

Methods and assessments:

**Visit Procedures (See Table 1)**

We will keep a subject screening log and a subject enrollment log.

In case of any premature discontinuation of the trial, we will make every effort to call in the subject for a last visit. We will specify primary reason (adverse event, non-compliance with protocol or other) for discontinuation in the CRF. If the subject is not able to attend, we will complete the End of Trial Form and fill in the Drug Accountability Form.

**Protocol**:

This research study involves 6 visits: two screening visits and 4 study visits. Subjects who meet the inclusion criteria will be invited to enroll in the study. Parental **consent** will be obtained before any subject is enrolled in the study. In addition to the parental consent, minors from age 10 to 15 years will read and sign an **assent form**. Older adolescents (16 and 17) will read and sign the same consent form as their parents signed. This ensures that all participants fully understand the requirements of the study and are competent to participate in it.Subjects will undergo a 3-month **run-in period** to screen for complications, to update patient education, and to assess subject’s compliance with study protocol. Patients will be placed on detemir and aspart with no adjunctive metformin or placebo during the run-in period. (Please see a detailed explanation of the run-in phase under Trial Design above). All participants will be instructed on the treat-to-target regimen and be given a **summary sheet** to familiarize themselves with the regimen. Treat-to-target regimen will only commence at the end of the run-in phase.

**1. VISIT I: START OF THE RUN-IN PERIOD AND SCREENING VISIT AT -3 MO**

*History:* To ensure eligibility for the study, we will take a detailed history of subject’s medical condition. This history will focus on patient’s current medical diagnosis, past medical and birth history, social history. We will document any family history of type 1 or type 2 diabetes, obesity, and related comorbidities. We will also document subject’s medications, supplements, diet and exercise plan.

*Physical Examination:* All participants will undergo a detailed physical examination at baseline, and at 3 month intervals throughout the duration of the study. The aims of the physical examination will be (1) to rule out medical conditions that could present with obesity, such as Cushing’s syndrome, severe hypothyroidism, and Prader Willi syndrome; (2) to identify complications of diabetes; (3) to identify dermatologic features associated with insulin resistance, such as acanthosis nigricans, and skin tags; (4) to document appropriate Tanner stages in all participants.

*Screening Laboratory Test:* All participants will undergo a screening study consisting of serum creatinine, liver function tests, thyroid function tests, and celiac antibody panel. Subjects with abnormal liver or kidney function tests will not be enrolled in the study. Subjects with abnormal thyroid function test will be treated and will be enrolled when euthyroid. Subjects with abnormal celiac antibody panel will be enrolled after a consultation with a gastroenterologist.

*Urine Sample:* A spot urine sample will be collected for microalbumin and creatinine at baseline. Urine pregnancy test will be obtained on all female participants and will be repeated at baseline, +3mo, and + 6mo.

*Run-in phase:* All participants will be transitioned to detemir and aspart, without instituting treat-to-target regimen, or adding metformin or placebo. (Please see a detailed explanation of the run-in phase under Trial Design above).

**2. VISIT II: REVIEW OF RUN-IN PERIOD GLYCEMIA AND NUTRITION COUNSELING AT -2 MO.**

All participants will return to the clinic one month after the start of the run-in phase for a brief physical examination, review of glucose data, and insulin dose adjustments. We will also review the protocol for patient-directed, treat-to-target regimen with the families.

*Nutrition Counseling*: All subjects will undergo nutrition counseling by a board certified dietician at -2, 0, +3, and at +6 months.

**3. VISIT III: BASELINE INTERVENTION STUDY VISIT AT O MO**

Following the screening study, qualified subjects will return to the Children’s Diabetes Service in the morning, after an overnight fast for initial intervention study blood draw. Anthropometric measurements: height, weight, waist circumference will be obtained. Vital signs and fasting glucose level will be checked. A venous catheter will be inserted in the antecubital fossa and blood sample will be drawn for fasting lipids, leptin, and 25-hydroxyvitamin D at baseline.

*Vital Signs:* Blood pressure and pulse will be measured using an electronic sphygmomanometer.

*Physical Examination:* Weight, height and waist circumference will be measured in all participants at baseline, 3 months and 6 months. Weight will be measured using an electronic scale with patient donning under-garments and a hospital gown. Values will be expressed to the nearest 0.1 kg. Height will be measured using a wall-mounted Harpenden stadiometer. Waist circumference will be measured at the level of the umbilicus, using a standard measuring tape. All measurements will be done in triplicates and the average taken. We will derive body mass index from the weight and height data.

*Blood samples:* At baseline, following an overnight fast, we will draw fasting blood samples for the following laboratory tests: lipid profile, leptin, adiponectin, hepatic function tests, serum creatinine, and 25-hydroxyvitamin D. These analytes will be redrawn at 3, 6 and 9 months. Only 2 teaspoons of blood will be drawn for these tests at each time point. All samples will be spun upon collection using a centrifuge and the serum collected and stored at -70 degrees until assay. Whole blood samples will be collected for HbA1c estimation.

Urine test: Urine pregnancy test will be obtained on all female participants and will be repeated at +3mo, and + 6mo.

**Procedure**

Following the blood draw, each subject will be started on a patient-directed, treat-to-target insulin regimen. All participants will be on multiple daily injections (MDI) consisting of once daily injection of long acting insulin analog, detemir, meal time insulin boluses with aspart, and a correction factor for insulin to ensure euglycemia between meals. All insulins will be administered using a flexpen device. For this study we will confirm the adequacy of each aspect of therapy by an ongoing monitoring system as described by Bode et al[59], and summarized below. We will use a ‘teach back’ protocol to ensure that patients understand the bolus insulin adjustment and correction factor plan.

Our goal for the participants is to maintain their blood glucose levels as shown below in the Table 2.

**Table 2. Summary of Expected Blood Glucose Levels at Different Times of the Day**

| Time | Before breakfast | Before lunch or dinner | Before bedtime | 2 hours after a meal | At 3AM |
| --- | --- | --- | --- | --- | --- |
| Expected Glucose level (mg/dL) | 90-120 | 80-130 | greater than 100 | less than 220 | greater than 100 |

**A. Bolus insulin dose adjustment plan:** All participants will be placed on treat-to-target regimen plus either metformin or placebo as described below. The insulin boluses for meals will be calculated from an insulin to carbohydrate ratio that would be predetermined by the endocrinology team for each patient. We will strive to maintain a basal : bolus insulin ratio of 1:1, i.e., each component will constitute 50% of the total daily dose of insulin. Our goal is to maintain a premeal blood glucose level of 90-130 mg/dL. To adjust premeal bolus doses, we will focus on the pattern of blood glucose levels before lunch, dinner, and bedtime. If the blood glucose levels at the same time of day (e.g., before dinner) are high **two days** in a row, we will increase the preceding premeal (e.g., before lunch) bolus by 10 percent. Conversely, we will decrease a preceding premeal bolus by 10% if a **single** low blood glucose is obtained before a meal. Changing this bolus dose the very next day will prevent a recurrence of hypoglycemia and thereby protect against the establishment of reduced hypoglycemic awareness[60]

**B. The correction factor or supplemental insulin dose adjustment plan:** The correction factor or supplemental insulin is the amount of fast-acting insulin that is added or subtracted from the insulin boluses to maintain euglycemia 2 hours after a meal as shown below in Table 3. The formula for the correction factor is (BG-X)/ISF, where BG is current blood glucose, X is ideal blood glucose for the patient, and ISF is insulin sensitivity factor. ISF will be calculated using the 1500 Rule, i.e., 1500 divided by the total daily dose of insulin. Our goal is to maintain a 2-hour post-prandial glucose level of <180 mg/L. To analyze the accuracy of the supplemental doses, we will look at every high blood glucose level and the level immediately following it. Specifically, if every high blood glucose level is followed by one in the target range, this indicates that the patient’s supplemental dose is correct. However, if most high blood glucose levels are followed by hypoglycemic levels, then the supplemental dose is too high. Conversely, if most high levels are followed by another high level, then the supplemental dose is too low. We will advise the participant to decrease or increase the sensitivity factor in the supplemental formula as needed to achieve the desired change in the blood glucose level as shown in Table 3.

**Table 3. Supplemental Insulin Dose Adjustment Plan**

| Prior meal Blood Glucose levels (mg/dL) | Current meal blood glucose level (mg/dL) | Supplemental Insulin dose (SID) |
| --- | --- | --- |
| greater than 130 | 80-130 | Interpretation: correct SID  Instruction: nothing to do |
| greater than 130 | less than 80 | Interpretation: SID is too high  Instruction: increase sensitivity factor by 10 |
| greater than 130 | greater than 130 | Interpretation: SID is too small  Instruction: decrease sensitivity factor by 10 |

**C. Basal Insulin adjustment plan:** Basal detemir injection will be given consistently at bedtime once a day. Subjects will titrate their long-acting insulin analog (detemir) dose every third day, to achieve a fasting plasma glucose of 90-120 mg/dL using the algorithm shown in Table 2 below. We will aim to maintain participants on a total daily **basal** insulin dose of 0.3-0.8 U/kg/day or higher if necessary, given once daily at bedtime. This will ensure relative normal inter-prandial glycemia and the achievement of our HbA1c targets.

**D. Metformin or Placebo:** All participants will be provided with a 3 month supply of metformin or placebo at baseline, +3, and +6mo. Subjects will be advised to start at one capsule daily for 2 weeks, and then increase to 2 capsules once daily for the remainder of the study duration.

**Table 2. Titration Algorithm for Long-acting insulin analog (Detemir)**

| **Average FPG of 3 consecutive days** | **Recommended Long-acting insulin analog dose adjustments** |
| --- | --- |
| <90 mg/dL (5.0 mmol/L) | -2 units of detemir |
| 90-120 mg/dl (5.0-6.7 mmol/L) | No adjustments |
| > 120 mg/dL (>6.7 mmol/L) | +2 units of detemir |

**Follow up phone calls:** Subjects will be followed with twice weekly phone calls in the first month of enrollment to assess compliance and to help calculate insulin titration doses and make necessary dose adjustments. After the first month of study, subjects will be contacted every 2 weeks until the end of the study. We will also instruct the families to call the diabetes line during the first week of treat to target regimen if any fingerstick blood glucose reading is <70 mg/dL or two readings of >250 mg/dL.

**Downloading of Glucometer reading:** Each subject will be provided with a glucometer. They will be instructed on how to download the glucose readings to a computer. They will be advised to download their glucometer readings on every third day and email or fax the downloaded data to a contact number or email address provided by the investigators for review and a follow up recommendation.

All participants will return to the clinic at +3 and +6 months for interim visits and at +9 months for study conclusion.

**4. VISIT IV: INTERVENTION STUDY INTERIM VISIT AT +3 MO**

Subjects will return to the clinic 3 months after starting on treat-to-target regimen plus either metformin or placebo for study follow up visit which will involve the following:

*Anthropometric measurements*: height, weight, waist circumference will be obtained. Vital signs and fasting glucose level will be checked. A venous catheter will be inserted in the antecubital fossa and blood sample will be drawn for fasting lipids, leptin, adiponectin, A1c, hepatic function tests, serum creatinine, and 25-hydroxyvitamin D.

*Vital Signs:* Blood pressure and pulse will be measured using an electronic sphygmomanometer.

*Anthropometric Measurements:* Weight, height and waist circumference will be measured in all participants. Weight will be measured using an electronic scale with patient donning under-garments and a hospital gown. Values will be expressed to the nearest 0.1 kg. Height will be measured using a wall-mounted Harpenden stadiometer. Waist circumference will be measured at the level of the umbilicus, using a standard measuring tape. All measurements will be done in triplicates and the average taken. We will derive body mass index from the weight and height data.

*Blood samples:* At 3 mo, following an overnight fast, we will draw fasting blood samples for the following laboratory tests: lipid profile, leptin, adiponectin, hemoglobin A1c, and 25-hydroxyvitamin D. These analytes will be redrawn at 6 and 9 months. Only 2 teaspoons of blood will be drawn for these tests at each time point. All samples will be spun upon collection using a centrifuge and the serum collected and stored at -70 degrees until assay.

Urine pregnancy test will be obtained on all female participants and will be repeated at + 6mo.

*Review of Glucose Data:* A pediatric endocrinologist will review patient’s glucose log book and glucometer and make necessary adjustments to insulin doses.

*Nutrition Evaluation:* A registered dietician will meet with the patients and his/her family and review patient’s interim dietary and make needed recommendations.

**5. VISIT V: INTERVENTION STUDY VISIT AT +6 MO**

**Visit V is identical to visit IV.**

**6. VISIT VI: STUDY CONCLUSION AT +9 MO**

At study conclusion, all subjects will return to the clinic in a fasting state for a final vital signs assessment, anthropometric measurements, physical examination, review of dossettes (pill counters), and a fasting blood draw for lipid profile, leptin, adiponectin, hemoglobin A1c, hepatic function tests, serum creatinine, and 25-hydroxyvitamin D as detailed below:

Subjects will return to the clinic 9 months after starting on treat-to-target regimen plus either metformin or placebo for end of study visit which will involve the following:

*End of study History and Physical Examination*: This discharge history and physical examination will focus on excluding any adverse effects from the study, and to establish endpoint anthropometric data: height, weight, waist circumference, and body mass index.

*Physical Examination*: height, weight, waist circumference will be obtained. Vital signs and fasting glucose level will be checked. A venous catheter will be inserted in the antecubital fossa and blood sample will be drawn for fasting lipids, leptin, adiponectin, HbA1c, hepatic function tests, serum creatinine, and 25-hydroxyvitamin D.

*Vital Signs:* Blood pressure and pulse will be measured using an electronic sphygmomanometer.

*Physical Examination (procedure):* Weight, height and waist circumference will be measured in all participants. Weight will be measured using an electronic scale with patient donning under-garments and a hospital gown. Values will be expressed to the nearest 0.1 kg. Height will be measured using a wall-mounted Harpenden stadiometer. Waist circumference will be measured at the level of the umbilicus, using a standard measuring tape. All measurements will be done in triplicates and the average taken. We will derive body mass index from the weight and height data.

*Blood samples:* At 9 mo, following an overnight fast, we will draw fasting blood samples for the following laboratory tests: lipid profile, leptin, adiponectin, and 25-hydroxyvitamin D. Only 2 teaspoons of blood will be drawn for these tests at each time point. All samples will be spun upon collection using a centrifuge and the serum collected and stored at -70 degrees until assay. Whole blood samples will be collected for HbA1c estimation. We will also obtain hepatic function tests and serum creatinine to compare the results to the screening and monitoring hepatic and kidney function studies to determine if there has been any effect of metformin use on participants’ hepatic and renal functions. One teaspoon of blood will be collected for this additional study.

*Urine Sample:* A spot urine sample will be collected for microalbumin and creatinine at study conclusion.

*Review of Glucose Data:* A pediatric endocrinologist will review patient’s glucose log book and glucometer and make necessary adjustments to insulin doses. This will conclude the study.

*Nutrition Evaluation:* A registered dietician will meet with the patients and his/her family and review patient’s interim dietary and make needed recommendations.

**Assessments for Efficacy**

1. **Laboratory assessments**: Fasting serum samples will be drawn at baseline and every 3 monthly for the following analytes, lipid panel, leptin, adiponectin, and 25-hydroxyvitamin D. All samples will be spun in a centrifuge and the serum stored at -70 degrees in a freezer at the Division of Endocrinology, Department of Pediatrics, University of Massachusetts Medical School. Whole blood samples will be collected for HbA1c estimation. At the end of the study, all samples will be assayed simultaneously by the Umass Memorial Hospital Biochemistry laboratory. HbA1c, the primary efficacy parameter, will be assayed by the HPLC method. Its inter-assay variability is <1.5%, and intra-assay variability is <2.5%, with a normal range of 4.4.-6.0%. Lipid profile will be assayed by the Enzymatic method; leptin by Human Leptin ELISA kits developed by LINCOplex (Linco Research, St Louis, MO) (sensitivity 0.01 ng/mL, interassay coefficient of variation 5%; adiponectin by Human Adiponectin ELISA kits developed by B-Bridge International (San Jose, CA) (lower limit 0.02 ng/ml; interassay coefficient of variation 3.2%); and 25-hydroxyvitamin D by chemiluminescence assay. All remaining specimens will be disposed of 5 years after the study.
2. **Body mass index**: Using a wall-mounted stadiometer for height measurement, and an electronic scale for body weight measurement, these anthropometic measurements will be obtained at -3, 0, +3, +6 and +9 months. Measurements will be taken in triplicates and the average taken.
3. **Insulin dosing**: All participants will be on basal-bolus insulin regimen. Weekly weight-adjusted total daily doses of insulin will be collected on all participants to identify changes in insulin requirements during the study.

**Assessments for Safety**

**Hypoglycemia**: The major safety concern for this study, as in the general management of diabetes, is hypoglycemia. For this study, the following classification of hypoglycemia will be used: (a) **major**: hypoglycaemic event requiring a third party assistance, (b) **minor**: self measured plasma glucose level of ≤ 60 mg/dL, (c) **symptoms only**: self-measured plasma glucose of > 60 mg/dL or no measurement during the episode, (d) **nocturnal**: self-measured plasma glucose of ≤ 60 mg/dL occurring between 11 pm and 6 am. Hypoglycemia will be monitored by self-measured plasma glucose by checking blood glucose before meals, at bedtime, and at 3 a.m. We will evaluate the glycemic profile every third day by reviewing the data sent in to our office by the participants’ families via fax or email. Insulin dose adjustments, and other appropriated suggestions will be made based on these data. We will ensure that participants maintain a bedtime blood glucose level of >100 mg/dL before going to sleep. It is the standard of care for patients to check their blood sugar level before bed and if it is less than 100 mg/dL to eat a snack and recheck their blood sugar to make sure that it is over 100 before going to bed.

Participants will also check 3AM blood glucose twice weekly and also on 2 consecutive nights following changes to their long-acting insulin dose.

**Renal and liver function tests**: We will obtain liver function studies, and creatinine levels at the beginning and at the end of the study.

We will monitor **weight, body mass index, and vital signs** at each visit.

**Subject Compliance**

We will assess subject compliance with the study in 3 ways: a), From information downloaded from the meter on every 3^rd^ day, (b), Dossette, (c), Contact with parents. There will be 2 criteria for compliance: (i) at least 6 distinct data downloads from the meter every month; and, (ii) less than 25% residual pills in the dossette at each visit.

g. Is any aspect of this research study being conducted in the Medical School or a non-UMMMC facility? If yes, please explain.

No.

h. Will hospitalization be required as part of this research study?

| Yes |  | No | X |
| --- | --- | --- | --- |

If yes, how long will subjects be hospitalized?

i. Will there be any material inducements or recruitment incentives given to research staff or research subjects as part of this research study? (e.g., direct payments, free hospitalization, care)

| YesX |  | No |  |
| --- | --- | --- | --- |

If yes, explain how much, the pay schedule, or any partial payments that will be given.

Each participant will receive $120 for the entire study. This payment will be made at the rate of $20 per visit for the 6 visits. This money will be issued as a gift card to a bookstore at each of the 6 visits.

*The committee is exceedingly sensitive to the threat of coercion that can stem from excessive compensation for participation in research. The IRB recommends hourly payments of $20/hr for every hour (or fraction thereof) the subject is involved in the study. This should include time in the hospital or clinic that is solely for the study, travel time, and time spent recovering from a procedure or an anesthetic agent used for a procedure. Time that the subject is unable to perform his/her routine activities of daily living due to study related issues should be included in this time. (Time required to perform multiple minor tasks should be lumped together; that is, filling out a questionnaire that takes 15 minutes on four different days constitutes one hour of labor, not four hours.) If reimbursed, cost of transportation ($0.35/mile), parking and meals should be noted. A bonus of up to $50 may be given for completion of a long term study or for studies that involve low risk but uncomfortable procedures (such as endoscopy, multiple blood samples for pharmacokinetic studies, gynecological examinations, etc).*

1. **DISCLOSURE OF CONFLICT OF INTEREST**

Investigators should disclose any financial arrangement they may have with a company whose product figures prominently in their research or financial arrangements they may have with company making a competing product. **The relationship should also be described in the informed consent documents**. In the case where the only relationship is that a company is sponsoring the research study, it is sufficient to prominently identify the sponsor on the front page of the consent form and to simply state “NONE” in the consent form under Conflict of Interest.

Is there a conflict of interest?

| Yes |  | No | X |
| --- | --- | --- | --- |

**6. RELATIONSHIP TO STANDARD THERAPY.**

Describe the standard therapy that patients would receive if not in the research study. Explain how this research intervention deviates from or replaces generally accepted standard therapy and justify the deviation.

Standard Treatment for Double Diabetes:

There is no consensus on the modalities of treatment for double diabetes in children and adolescents. Most endocrinologists would treat these individuals with insulin only. However, recent reports[43] now suggest that overweight adolescents with type 1 diabetes who are going through puberty would benefit from adjunctive therapy with agents that counteract the insulin resistance brought on by increased adiposity. Other studies have shown that patients with type 2 diabetes who possess diabetes-associated autoantibodies lose their beta cells faster than those without autoantibodies and thus will be dependent on insulin therapy sooner. Therefore, our study does not deviate from the emerging method of treating patients with double diabetes. Even though we cannot say that treat-to-target regimen is standard of care in children, the blood glucose targets we are using for age are defined by the American Diabetes Association. The only difference is that we have a dedicated staff that will follow up with the family to ensure compliance with the guidelines. Determir and aspart are standard of care.

RESEARCH INTERVENTION STUDY:

Eligible candidates will be required to receive either placebo or metformin 1000 mg daily. The follow up schedule and monitoring regimen are the same for other patients with diabetes.

The Research Study Protocol deviates from the Standard management of Double Diabetes:

- Quarterly blood draws for lipids, leptin, adiponectin, serum creatinine, hepatic function tests and 25OHD. The standard of care for blood draws is yearly blood draw for lipids, thyroid function tests, celiac antibodies, 25OHD, and quarterly blood draws for HbA1c.

**7. DESCRIBE THE POTENTIAL BENEFITS OF THIS PROJECT.**

1. Include hoped-for benefit to society, to the group of subjects or to individual subjects.
2. Address the risk/benefit ratio of the study. If there are no direct subject benefits, this should be stated.

Benefit to Society:

This study will help to determine the efficacy of metformin therapy in reducing both A1c levels and body mass index in patients with double diabetes. It is our hope that the findings from this study would help in the development of new treatment regimen for double diabetes.

Benefit to subjects:

Subjects may benefit directly by experiencing an improvement in their glycemic control, which might translate to a reduction in their risk of diabetes complications. Those randomized to metformin arm of the trial may also benefit from the mild weight loss effect of metformin.

Risk/Benefit ratio of the study:

Apart from the initial blood draw for screening tests and subsequent blood draws every 3 mo for lipids, leptin, adiponectin, A1c, and 25OHD, the study has the same level of risk as the standard therapy for double diabetes. The risk benefit ratio is very low, i.e., the subjects are not subjected to any additional risk other than what they would experience during a standard treatment for double diabetes. Metformin therapy is generally safe and is widely used in the treatment of type 2 diabetes and polycystic ovarian syndrome in children and adolescents. It is the only oral drug approved by the Food and Drug Administration for use in the management of diabetes in children.

**8. DESCRIBE THE POTENTIAL RISKS TO SUBJECTS INCLUDE PSYCHOLOGICAL, ECONOMIC, LEGAL OR SOCIAL RISKS AS WELL AS PHYSICAL RISKS.**

**Include the following information:**

a. Estimate likelihood of occurrence, severity, and duration. If generally accepted quantitative estimates are available based on previous data, these should be stated. Otherwise, qualitative estimates such as “rare”, “occasionally", or “frequently” may be used. *The committee needs scientific information about drug/device side effects so as to best judge the pros and cons of the study.* ***Do not simply cut and paste the consent form “Risk” section into this part of the protocol.***

Risks associated with study procedure:

Some children and adolescents may become embarrassed when given a physical examination or asked detailed questions about their diabetes. Some may be bothered by having blood drawn due to fear of needles or concern about pain. A numbing cream used to minimize pain can cause a slight swelling at the application site. There is a minor risk of infection at the site of the needle stick. In about 1 in 20 cases, a child may faint or become sick to the stomach at the sight of a needle or when blood is drawn. There may be some discomfort or bruising at the site of the needle stick. To alleviate the pain of needle stick, EMLA cream will be provided for subjects who wish to numb the site of blood draw before the needle stick.

Risks associated with medications:

1. Metformin: As a result of participating in this study, subjects randomized to the metformin arm will receive metformin 1000 mg once daily by mouth for 9 months. This dose is within the range of normal starting dose of metformin for pediatric patients with either diabetes or polycystic ovarian syndrome. Common side effects associated with metformin therapy include nausea, loss of appetite, diarrhea, gas, and a metallic taste. These usually subside with time and do not necessitate discontinuing the medication. Lactic acidosis is a rare but potentially serious side effect that is more likely to occur in individuals with heart, kidney, or liver failure. Lactic acidosis is very rare in children with diabetes.
2. Insulin: Hypoglycemia is a recognized risk of insulin therapy. However, all our participants would have been on insulin therapy for at least 6 months prior to being enrolled in our study. Thus, they have the knowledge and skills to recognize and manage hypoglycemia. We will also conduct a session on our titration regimen during the Run-in phase to familiarize participants with the protocol and possible side effects such as hypoglycemia.

Treat to target regimen and oral medications will not be initiated simultaneously. Our protocol is to initiate and familiarize the patients with all aspects of treat to target regimen during the run-in phase of the study without adding metformin or placebo. Thereafter, we will introduce the oral agents. Therefore, subjects will have ample time to work with treat to target regimen before receiving oral agents.

We do not anticipate an increased frequency or risk of hypoglycemia with the treat to target regimen because treat to target regimen is not a unidirectional model of care. It is based on a bidirectional flexible model that allows the patient to subtract or add 1-2 units to his/her long acting insulin dose depending on his 3-day average fasting blood glucose level. We have also built in a close follow up and monitoring plan for this study. For example, the frequent phone calls during which we will review finger stick blood glucose readings with the family and the fact that the families will fax or email finger stick blood glucose readings to us twice weekly will ensure very close monitoring of blood glucose excursions throughout the study duration. We will also ensure that participants maintain a bedtime blood glucose level of >100 mg/dL before going to sleep. Participants will also check 3AM blood glucose twice weekly and also on 2 consecutive nights following changes to their long-acting insulin dose. We will also instruct the families to call the diabetes line during the first week of treat to target regimen if any fingerstick blood glucose reading is <70 mg/dL or two readings of >250 mg/dL.

We also do not anticipate the adjunctive metformin therapy to cause hypoglycemia as we will be titrating basal insulin doses to the prevailing glycemia, and will reduce the dose of basal insulin if there is evidence of incipient hypoglycemia.

Finally, we have included a Hypoglycemia Monitoring Table to enable participants document their low sugar reactions in an effective way so we can address these reactions promptly.

b. Explain what steps will be taken to protect against its occurrence, minimizing the harm, methods for early detection of harm, and what procedures will be followed to avoid serious injury (e.g. withdraw from study or dose reduction).

Study Safety Officer: Samir Malkani, MD.

As the safety officer Dr Malkani will monitor participants in the study for adverse events related to the study drug, and make recommendations regarding removing subjects from the study or terminating the study if the incidence of adverse effects is significant.

He will review unblinded study data every month for the first 3 months of the study, and then every three months thereafter with regards to assignment of subjects to the study arms, prevalence of gastrointestinal side effects (including nausea, diarrhea and abdominal discomfort), unusual weight loss (>10% of body weight in 3 months), admissions to hospital or elevation of liver enzymes to >3 times above the normal limit. He will also review all other SAEs. This information will be forwarded to him by the study coordinator, and unblinding information by Investigational Drug Services which is responsible for blinding the study drug. He will perform this review every 3 months until the completion of the study. If an issue is found, he will report this promptly to the PI and will jointly issue an adverse effects or SAE report (as the case may be) with the PI to the UMass IRB and the study sponsor, Novo Nordisk Inc.

**Other steps to minimize harm:**

Baseline vital sign check will be obtained before the study. Emergency resuscitation equipment are available in the Pediatric Phlebotomy Room should resuscitation be necessary.

Risks of anxiety, infection, pain and bruising due to blood draws will be minimized by making sure that the child is relaxed, using universal precautions to avoid infection, using EMLA cream to numb the site of the needle stick, and making sure all staff are trained in pediatric phlebotomy technique, and are experienced in working with children.

We will check liver function tests in all subjects before starting metformin. We will discontinue metformin if subjects experience any illnesses leading to vomiting or diarrhea or dehydration. Metformin should also be discontinued if patient is scheduled to undergo radiologic tests requiring a contrast agent.

c. Explain whether or not these risks are from a procedure performed with the **intent** and **reasonable prospect** of yielding **direct** health related benefit to the subject.

All of the above risks are similar to the risks associated with the standard management of diabetes in children and adolescents and are done with the intent and reasonable prospect of yielding direct health related benefit to the subjects.

d. Do you, as the PI, have equipoise regarding the study? That is, are you comfortable with the risks in relationship to the knowledge gained? If the study involves randomization, do you believe in the equality of the treatment arms?

I have absolute equipoise regarding this study. I believe that the risks associated with the study are outweighed by the potential benefit of demonstrating A1c reduction by adjuvant metformin therapy in children and adolescents with double diabetes.

**9. CONFIDENTIALITY CONSIDERATIONS: EXPLAIN STEPS THAT WILL BE TAKEN TO INSURE THE CONFIDENTIALITY OF INFORMATION THAT IS OBTAINED IN THE COURSE OF THIS RESEARCH PROJECT. INCLUDE THE FOLLOWING:**

a. How will identifiers be used?

This study will comply with all HIPAA guidelines regarding confidentiality of patients’ data. Though, this study will be done in parallel with clinical evaluation of patients coming for diabetes management, effort will be made to protect patients’ information for this study, by providing each subject with a study identification number. The personal identifiers will be kept separately from patient files in a secure, locked location at the Department of Pediatrics to assure confidentiality. Only the PI and study staff member will have access to these files.

b. Where will data be stored?

The personal identifiers and study data will be kept separately from patient files in a secure, locked location in the research office of the Division of Endocrinology in the Department of Pediatrics, UMMS, to ensure confidentiality.

c. Who will have access to the data (please include UMMS IRB and their representatives as someone who may have access to this information)?

The research nurse coordinator will record data. Electronic files will be stored at the PI’s desk top computer located at the department of Pediatrics, University of Massachusetts Medical School. Only the PI, co-investigators and study staff members, UMMS IRB, and their representatives will have access to these files. PI’s computer is password protected.

d. When will the data/specimens be destroyed?

Specimens and data will be kept for five years after study end, after which time they will be destroyed.

1. In the future, might other use be made of specimens collected as part of the research? If yes, please describe.

No.

**10. ECONOMIC CONSIDERATIONS:**

1. In the course of this research project, might the subjects experience any additional expenses as a result of study participation? This includes both out-of-pocket costs and expenses that might not be covered by medical insurance.

| Yes |  | No | X |
| --- | --- | --- | --- |

If yes, please explain and justify.

b. Please explain potential increase in standard hospital costs if any.

None

**11. DESCRIBE THE CHARACTERISTICS OF THE SUBJECT POPULATION.**

a. The subject population includes:

ADULTS X CHILDREN X

b. Is the subject population restricted in respect to any of the following characteristics?

| Please “x” those that apply | Yes | No |
| --- | --- | --- |
| Age Range | X |  |
| Health Status | X |  |
| Gender |  | X |
| Racial/Ethnic composition |  | X |

If you responded **YES** to any of the above, include a clear rationale for this restriction.

Our study population includes children and adolescents of 10-18 years old with double diabetes.

**12. WILL THE STUDY POPULATION SPECIFICALLY INCLUDE A POPULATION OF SUBJECTS CONSIDERED “VULNERABLE”? VULNERABLE POPULATIONS ARE CHILDREN, MENTALLY IMPAIRED, PREGNANT WOMEN, PRISONERS, OR FETUSES.**

| Yes | X | No |  |
| --- | --- | --- | --- |

If yes, please explain.

This study is designed to determine the effects of metformin therapy on A1c and BMI in children and adolescents of 10-18 years of age who have double diabetes. Our objective is to determine the efficacy of HbA1c reduction in these patients.

**13. WHAT IS THE SOURCE OF THE SUBJECT POPULATION?**

Study participants will be recruited from the outpatient pediatric endocrinology program of the Children’s Medical Center, UMass Memorial Medical Center.

**14. EXPLAIN ANY STEPS TAKEN TO INSURE THAT THE SUBJECT POPULATION IS REPRESENTATIVE.**

This study is open to all children and adolescents of ages 10-18 years who fulfill the inclusion criteria, regardless of race, ethnicity or gender.

**15. HOW AND WHERE WILL SUBJECTS BE RECRUITED FOR THE STUDY? CONSULT THE** [**IRB Guidelines**](http://www.umassmed.edu/subjects/human/uploads/guidelines2004.doc) **FOR THE RESTRICTIONS ON RECRUITMENT OF EMPLOYEES, STUDENTS, AND INPATIENTS. ATTACH COPIES OF ALL RECRUITMENT MATERIALS TO BE USED AS PART OF THIS RESEARCH STUDY. THESE MATERIALS MUST BE APPROVED BY THE IRB BEFORE BEING USED.**

All subjects will be recruited from the outpatient pediatric endocrinology program of the UMass Memorial Medical Center.

Subjects will be identified as potentially eligible for the study a pediatric endocrinologist, who will review their medical records to ensure that the subject is eligible for the study. Once identified, the patient’s endocrinologist will discuss this study with the potential subject and his/her parent(s) or guardian(s). If they are interested in learning more, they will be contacted by a study physician to go over details of the study to determine whether they are interested in participating in the study.

**16. WILL** [Protected Health Information](http://www.umassmed.edu/subjects/human/uploads/phi.doc) **(PHI) BE USED AS PART OF THIS RESEARCH STUDY? PLEASE VISIT OUR** [**WEBSITE**](http://www.umassmed.edu/subjects/human/hipaa/) **FOR MORE INFORMATION ABOUT PHI OR THE HEALTH INSURANCE PORTABILITY AND ACCOUNTABILITY ACT (HIPAA).**

| Yes | X | No |  |
| --- | --- | --- | --- |

If **yes**, please answer the following questions.

1. **How and where will the PHI be accessed (i.e. meditech, database, medical records, another site)?**

The PHI will be accessed from the Meditech, Allscripts, and patient’s medical records only by the PI and members of the Metformin study staff.

1. **Will a subject’s PHI be accessed before the subject is enrolled in the study?**

PHI information may be accessed to ensure eligibility before enrolling subjects for the study.

1. **Please list the PHI to be used as part of this research study (i.e. name, DOB, medical record #).**

Name, DOB, Medical Record Number, diagnoses, age, sex, height, weight, laboratory studies.

**17. METHOD FOR OBTAINING INFORMED CONSENT**

a. Are you requesting a waiver of the requirement for obtaining consent?

| Yes |  | No | X |
| --- | --- | --- | --- |

If yes, please justify the request in the box below. Consent may be waived if research is minimal risk; the waiver does not adversely affect the subject **and the research could not practically be carried out without the waiver**. Your justification must address these issues.

**Do not complete the following questions if you are requesting a waiver of informed consent.**

**18. WILL VERBAL CONSENT BE OBTAINED?**

| Yes |  | No | X |
| --- | --- | --- | --- |

If **yes**, will an unsigned “fact sheet” be given to subjects before verbal consent is obtained?

| Yes |  | No |  |
| --- | --- | --- | --- |

If **yes**, please provide a copy of the “fact sheet”.

**19. WILL A SIGNED CONSENT FORM BE REQUIRED?**

| Yes | X | No |  |
| --- | --- | --- | --- |

1. **AS A GROUP, ARE THESE SUBJECTS EXPECTED TO BE COMPETENT TO GIVE CONSENT FOR THEMSELVES?**

| Yes |  | No | X |
| --- | --- | --- | --- |

If **no**, please explain why and how consent will be obtained.

- Parental consent will be obtained before any subject is enrolled in the study. In addition to the parental consent, minors from age 10 to 15 years will read and sign an assent form. Older adolescents (16 and 17) will read and sign the same consent form as their parents signed. This ensures that all participants fully understand the requirements of the study and are competent to participate in it.

**21. EXPLAIN THE CIRCUMSTANCES UNDER WHICH CONSENT WILL BE OBTAINED. HOW WILL YOU INSURE THAT POTENTIAL SUBJECTS HAVE ADEQUATE TIME TO CONSIDER THEIR OPTIONS, AND THAT POSSIBLE COERCION IS MINIMAL?**

The investigators will approach eligible minors and their families to discuss the study. They will be provided with information about the study and given at least 3 hours to review the materials before agreeing to participate. Minors will be given the opportunity to discuss the study alone with the investigators. If during this interview, the minor expresses strong concerns about or states that he or she is not interested in participating in the study, he or she will not be enrolled in the study.

Consent and assent will be obtained by study physicians. Permission for enrolled children will be obtained from each child’s parent. Children will sign assent forms.

**22. IF THE SUBJECT POPULATION INCLUDES MINORS, AND SIGNED CONSENT WILL BE OBTAINED, WILL AN ASSENT FORM BE USED AS PART OF THE CONSENTING PROCESS? CONSULT** [**IRB Guidelines**](http://www.umassmed.edu/subjects/human/uploads/guidelines2004.doc) **FOR INFORMATION ABOUT CHILDREN IN RESEARCH STUDIES.**

| Yes | X | No Minors enrolled |  | Verbal consent requested |  |
| --- | --- | --- | --- | --- | --- |

**NOTE: In general, it is expected that minors from age 12 to 15 will read and sign an assent form. Older adolescents (16 and 17) will usually read and sign the same consent form as the parents signed. The** [**assent form template**](http://www.umassmed.edu/subjects/human/forms.cfm) **is available on our website.**

**22. IF YES, PLEASE EXPLAIN WHO WILL APPROACH THE MINORS AND HOW AND WHERE THE ASSENTING PROCUDURE WILL TAKE PLACE.**

Potentially eligible minors will be identified for Metformin study by their pediatric endocrinologist. A study physician will discuss the study with the minor and his/her parent or legal guardian, at the same setting.

**SECTION VI**

**CERTIFICATION OF APPROVAL**

**PI Name: Benjamin U. Nwosu, MD**

**DELEGATION OF ROLES/RESPONSIBILITIES*: Checklist/Signature List**

| **Please type**  **Name and Credentials** | **Role*** | **Signature** | **Department/Campus** | **Delegation of responsibilities: Please use key** in box below to summarize your study activities and place an “x” in the appropriate column** | | | | | | | | | |
| --- | --- | --- | --- | --- | --- | --- | --- | --- | --- | --- | --- | --- | --- |
|  |  |  |  | **A** | **B** | **C** | **D** | **E** | **F** | **G** | **H** | **I** | **J** |
| Mary M. Lee, MD | 1 |  | Pediatrics | X | X | X |  |  |  |  |  |  |  |
| Karen Cullen, RN, CDE | 2 |  | Pediatrics | X |  |  | X | X | X | X | X | X |  |
| Louise Maranda, PhD | 1 |  | Pediatrics |  |  |  |  |  |  |  |  |  | X |
| Leslie Soyka, MD  Amanda Angelescu, MD  Olga T. Hardy, MD  Penny Kadmon, MD  Lisa Greenman, RD*  Michael Stalvey, MD | 1  1  1  1  4  1 |  | Pediatrics  Pediatrics  Pediatrics  Pediatrics  Pediatrics  Pediatrics | X  X  X  X  X | X  X  X  X  X | X  X  X  X  X |  |  |  |  |  |  | X |
|  |  |  |  |  |  |  |  |  |  |  |  |  |  |
|  |  |  |  |  |  |  |  |  |  |  |  |  |  |
|  |  |  |  |  |  |  |  |  |  |  |  |  |  |

***Roles:** (choose appropriate # below)

| **1. Sub or Co-Investigator** | **2. Study Nurse Coordinator** | **3. Study Coordinator** | **4. Other:** |
| --- | --- | --- | --- |

****Delegation of Responsibility Codes: (choose all that apply)**

| **A. Consent Subjects** | **F. Maintain Regulatory Documents** |
| --- | --- |
| **B. Take Medical History** | **G. CRF Completion and Query Resolution** |
| **C. Conduct Physical Exam** | **H. SAE/AE Monitoring/Reporting** |
| **D. Phlebotomy** | **I. IRB Communications and Continuing Review** |
| **E. Monitor Vital Signs/Nursing Assessment** | **J. Other (explain): Statistical Analysis and Study Design; Nutrition counseling*** |

Although the Principal Investigator is ultimately responsible for every element of study activity, this form serves to clarify to whom the PI has delegated specific study activities and responsibilities.

|  |  |  |  |
| --- | --- | --- | --- |

REFERENCES

1. Hedley AA, Ogden CL, Johnson CL, Carroll MD, Curtin LR, Flegal KM: Prevalence of overweight and obesity among US children, adolescents, and adults, 1999-2002. Jama 2004;291:2847-2850.

2. Flegal KM, Wei R, Ogden C: Weight-for-stature compared with body mass index-for-age growth charts for the United States from the Centers for Disease Control and Prevention. Am J Clin Nutr 2002;75:761-766.

3. Himes JH, Dietz WH: Guidelines for overweight in adolescent preventive services: recommendations from an expert committee. The Expert Committee on Clinical Guidelines for Overweight in Adolescent Preventive Services. Am J Clin Nutr 1994;59:307-316.

4. Flodmark CE, Lissau I, Moreno LA, Pietrobelli A, Widhalm K: New insights into the field of children and adolescents' obesity: the European perspective. Int J Obes Relat Metab Disord 2004;28:1189-1196.

5. Ogden CL, Flegal KM, Carroll MD, Johnson CL: Prevalence and trends in overweight among US children and adolescents, 1999-2000. Jama 2002;288:1728-1732.

6. Ogden CL, Carroll MD, Curtin LR, McDowell MA, Tabak CJ, Flegal KM: Prevalence of overweight and obesity in the United States, 1999-2004. Jama 2006;295:1549-1555.

7. Troiano RP, Flegal KM, Kuczmarski RJ, Campbell SM, Johnson CL: Overweight prevalence and trends for children and adolescents. The National Health and Nutrition Examination Surveys, 1963 to 1991. Arch Pediatr Adolesc Med 1995;149:1085-1091.

8. Must A, Strauss RS: Risks and consequences of childhood and adolescent obesity. Int J Obes Relat Metab Disord 1999;23 Suppl 2:S2-11.

9. Daniels SR, Arnett DK, Eckel RH, Gidding SS, Hayman LL, Kumanyika S, Robinson TN, Scott BJ, St Jeor S, Williams CL: Overweight in children and adolescents: pathophysiology, consequences, prevention, and treatment. Circulation 2005;111:1999-2012.

10. Ebbeling CB, Pawlak DB, Ludwig DS: Childhood obesity: public-health crisis, common sense cure. Lancet 2002;360:473-482.

11. Williams J, Wake M, Hesketh K, Maher E, Waters E: Health-related quality of life of overweight and obese children. Jama 2005;293:70-76.

12. Schwimmer JB, Burwinkle TM, Varni JW: Health-related quality of life of severely obese children and adolescents. Jama 2003;289:1813-1819.

13. Kaufman F: 'Double diabetes' in young people and how to treat it. Diabetes Voice 2006;51:19-22.

14. Pickup JC, Williams, G.: Textbook of Diabetes. ed 3rd, Blackwell Publishing, 2002.

15. Pozzilli P, Guglielmi C: Double diabetes: a mixture of type 1 and type 2 diabetes in youth. Endocr Dev 2009;14:151-166.

16. Reinehr T, Schober E, Wiegand S, Thon A, Holl R: Beta-cell autoantibodies in children with type 2 diabetes mellitus: subgroup or misclassification? Arch Dis Child 2006;91:473-477.

17. Libman IM, Becker DJ: Coexistence of type 1 and type 2 diabetes mellitus: "double" diabetes? Pediatr Diabetes 2003;4:110-113.

18. Double Diabetes Summary. In, Children With Diabetes, 2009.

19. Variation and trends in incidence of childhood diabetes in Europe. EURODIAB ACE Study Group. Lancet 2000;355:873-876.

20. CDC: Epidemiology of Type 1 and Type 2 Diabetes Mellitus Among North American Children and Adolescents. In, Center For Disease Control and Prevention, 2008.

21. Economic costs of diabetes in the U.S. In 2007. Diabetes Care 2008;31:596-615.

22. Petitti DB, Klingensmith GJ, Bell RA, Andrews JS, Dabelea D, Imperatore G, Marcovina S, Pihoker C, Standiford D, Waitzfelder B, Mayer-Davis E: Glycemic Control in Youth with Diabetes: The SEARCH for Diabetes in Youth Study. J Pediatr 2009;155:668-672.

23. Type 2 diabetes in children and adolescents. American Diabetes Association. Diabetes Care 2000;23:381-389.

24. Kibirige M, Metcalf B, Renuka R, Wilkin TJ: Testing the accelerator hypothesis: the relationship between body mass and age at diagnosis of type 1 diabetes. Diabetes Care 2003;26:2865-2870.

25. Wilkin TJ: The accelerator hypothesis: a review of the evidence for insulin resistance as the basis for type I as well as type II diabetes. Int J Obes (Lond) 2009.

26. Liu LL, Lawrence JM, Davis C, Liese AD, Pettitt DJ, Pihoker C, Dabelea D, Hamman R, Waitzfelder B, Kahn HS: Prevalence of overweight and obesity in youth with diabetes in USA: the SEARCH for Diabetes in Youth Study. Pediatr Diabetes 2009.

27. Erbey JR, Kuller LH, Becker DJ, Orchard TJ: The association between a family history of type 2 diabetes and coronary artery disease in a type 1 diabetes population. Diabetes Care 1998;21:610-614.

28. Williams KV, Erbey JR, Becker D, Orchard TJ: Improved glycemic control reduces the impact of weight gain on cardiovascular risk factors in type 1 diabetes. The Epidemiology of Diabetes Complications Study. Diabetes Care 1999;22:1084-1091.

29. Effect of intensive diabetes treatment on the development and progression of long-term complications in adolescents with insulin-dependent diabetes mellitus: Diabetes Control and Complications Trial. Diabetes Control and Complications Trial Research Group. J Pediatr 1994;125:177-188.

30. de Boer IH, Sibley SD, Kestenbaum B, Sampson JN, Young B, Cleary PA, Steffes MW, Weiss NS, Brunzell JD: Central obesity, incident microalbuminuria, and change in creatinine clearance in the epidemiology of diabetes interventions and complications study. J Am Soc Nephrol 2007;18:235-243.

31. Effect of intensive diabetes management on macrovascular events and risk factors in the Diabetes Control and Complications Trial. Am J Cardiol 1995;75:894-903.

32. Jones KL: Role of obesity in complicating and confusing the diagnosis and treatment of diabetes in children. Pediatrics 2008;121:361-368.

33. Rosenbloom AL, Wheeler L, Bianchi R, Chin FT, Tiwary CM, Grgic A: Age-adjusted analysis of insulin responses during normal and abnormal glucose tolerance tests in children and adolescents. Diabetes 1975;24:820-828.

34. Caprio S, Tamborlane WV: Metabolic impact of obesity in childhood. Endocrinol Metab Clin North Am 1999;28:731-747.

35. Miller J SJ, Rosenbloom AL.: Pediatric Endocrinology. ed 5th, New York, Informa Healthcare USA, Inc., 2007.

36. Miller J, Silverstein, J.H., Rosenbloom, A.L.: Pediatric Endocrinology. ed 5th, New York, Informa Healthcare USA, Inc., 2007.

37. Mortensen HB, Robertson KJ, Aanstoot HJ, Danne T, Holl RW, Hougaard P, Atchison JA, Chiarelli F, Daneman D, Dinesen B, Dorchy H, Garandeau P, Greene S, Hoey H, Kaprio EA, Kocova M, Martul P, Matsuura N, Schoenle EJ, Sovik O, Swift PG, Tsou RM, Vanelli M, Aman J: Insulin management and metabolic control of type 1 diabetes mellitus in childhood and adolescence in 18 countries. Hvidore Study Group on Childhood Diabetes. Diabet Med 1998;15:752-759.

38. Kahn BB, Flier JS: Obesity and insulin resistance. J Clin Invest 2000;106:473-481.

39. Chalew SA, Gomez R, Butler A, Hempe J, Compton T, Mercante D, Rao J, Vargas A: Predictors of glycemic control in children with type 1 diabetes: the importance of race. J Diabetes Complications 2000;14:71-77.

40. Gallegos-Macias AR, Macias SR, Kaufman E, Skipper B, Kalishman N: Relationship between glycemic control, ethnicity and socioeconomic status in Hispanic and white non-Hispanic youths with type 1 diabetes mellitus. Pediatr Diabetes 2003;4:19-23.

41. Hamilton J, Cummings E, Zdravkovic V, Finegood D, Daneman D: Metformin as an adjunct therapy in adolescents with type 1 diabetes and insulin resistance: a randomized controlled trial. Diabetes Care 2003;26:138-143.

42. Meyer L, Bohme P, Delbachian I, Lehert P, Cugnardey N, Drouin P, Guerci B: The benefits of metformin therapy during continuous subcutaneous insulin infusion treatment of type 1 diabetic patients. Diabetes Care 2002;25:2153-2158.

43. Sarnblad S, Kroon M, Aman J: Metformin as additional therapy in adolescents with poorly controlled type 1 diabetes: randomised placebo-controlled trial with aspects on insulin sensitivity. Eur J Endocrinol 2003;149:323-329.

44. Howlett HC, Bailey CJ: A risk-benefit assessment of metformin in type 2 diabetes mellitus. Drug Saf 1999;20:489-503.

45. Mehnert H: Metformin, the rebirth of a biguanide: mechanism of action and place in the prevention and treatment of insulin resistance. Exp Clin Endocrinol Diabetes 2001;109 Suppl 2:S259-264.

46. Effect of intensive blood-glucose control with metformin on complications in overweight patients with type 2 diabetes (UKPDS 34). UK Prospective Diabetes Study (UKPDS) Group. Lancet 1998;352:854-865.

47. Jones KL, Arslanian S, Peterokova VA, Park JS, Tomlinson MJ: Effect of metformin in pediatric patients with type 2 diabetes: a randomized controlled trial. Diabetes Care 2002;25:89-94.

48. Desmangles J BJ, Shine B, Quattrin T.: Is Metformin a useful adjunct to insulin therapy in adolescents with type 1 diabetes in poor control? In Endocrine Society Meeting. 2000.

49. Walravens PA CP, Klingensmith GJ, Essison M, Cornell C, Monahan K.: Low Dose Metformin in adolescents type 1 diabetes mellitus: a double blind, controlled study. In American Diabetes Association 60th Scientific Sessions. 2000.

50. Abdelghaffar S, Attia AM: Metformin added to insulin therapy for type 1 diabetes mellitus in adolescents. Cochrane Database Syst Rev 2009:CD006691.

51. Scarlett JA, Gray RS, Griffin J, Olefsky JM, Kolterman OG: Insulin treatment reverses the insulin resistance of type II diabetes mellitus. Diabetes Care 1982;5:353-363.

52. Yki-Jarvinen H, Koivisto VA: Natural course of insulin resistance in type I diabetes. N Engl J Med 1986;315:224-230.

53. Intensive blood-glucose control with sulphonylureas or insulin compared with conventional treatment and risk of complications in patients with type 2 diabetes (UKPDS 33). UK Prospective Diabetes Study (UKPDS) Group. Lancet 1998;352:837-853.

54. Davies M, Storms F, Shutler S, Bianchi-Biscay M, Gomis R: Improvement of glycemic control in subjects with poorly controlled type 2 diabetes: comparison of two treatment algorithms using insulin glargine. Diabetes Care 2005;28:1282-1288.

55. Blonde L, Merilainen M, Karwe V, Raskin P: Patient-directed titration for achieving glycaemic goals using a once-daily basal insulin analogue: an assessment of two different fasting plasma glucose targets - the TITRATE study. Diabetes Obes Metab 2009;11:623-631.

56. Aldasouqi SA, Duick DS: Safety issues on metformin use. Diabetes Care 2003;26:3356-3357.

57. Faichney JD, Tate PW: Metformin in type 1 diabetes: is this a good or bad idea? Diabetes Care 2003;26:1655.

58. Misbin RI, Green L, Stadel BV, Gueriguian JL, Gubbi A, Fleming GA: Lactic acidosis in patients with diabetes treated with metformin. N Engl J Med 1998;338:265-266.

59. Bode BW, Davidson, P.C. (ed): The Insulin Pump Therapy Book. Los Angeles, Minimed Technologies, 1995.

60. Cryer PE: Hypoglycemia begets hypoglycemia in IDDM. Diabetes 1993;42:1691-1693.
