# Supplementary material for: A Randomized, Double-Blind, Placebo-Controlled Trial of Adjunctive Metformin Therapy in Overweight/Obese Youth with Type 1 Diabetes
Source: PLoS One. 2015 Sep 14;10(9):e0137525. doi: 10.1371/journal.pone.0137525 (PMC4569440; doi:10.1371/journal.pone.0137525)
Supplement: S1 Amendment — (PDF) [file pone.0137525.s003.pdf]

University of Massachusetts
UMASS. Medical School

Human Subjects/IRB
Office of the Vice Provost for Research
University of Massachusetts Medical School
55 Lake Avenue North
Worcester, MA 01655-0002 USA
508.856.4261 (office) 508.856.5004 (fax)

September 26, 2011

Benjamin Nwosu M.D.
Pediatrics
University Campus
RE: CHANGES IN PROTOCOL DOCKET 13938

Dear Dr. Nwosu

The Committee for the Protection of Human Subjects in Research has approved the changes in your protocol entitled: Glycemic Control in Children and Adolescents with Double Diabetes: Trial of Optimized Insulin-Metformin Regimen

The changes requested are as follows:

Change subject population from 10~18 years to 10~20 years.

The revised consent form & new adult consent form, which you have submitted is satisfactory and approved copies, are enclosed.

Please Note: Amendments that alter any of the drugs used in this research study must be forwarded to Lucie LaJeunesse, the research pharmacist.

Thank you for your cooperation with the Committee.

Sincerely,

Michael Centola MHS, CIP
Manager, Institutional Review Board
